# Ca–Ag compounds in ethylene epoxidation reaction

**DOI:** 10.1080/14686996.2019.1655664

**Published:** 2019-08-14

**Authors:** Iryna Antonyshyn, Olga Sichevych, Alim Ormeci, Ulrich Burkhardt, Karsten Rasim, Sven Titlbach, Marc Armbrüster, Stephan A. Schunk, Yuri Grin

**Affiliations:** a Chemical Metals Science Department, Max-Planck-Institut für Chemische Physik fester Stoffe, Dresden, Germany; b hte GmbH, Heidelberg, Germany; c Faculty of Natural Sciences, Institute of Chemistry, Materials for Innovative Energy Concepts, Chemnitz University of Technology, Chemnitz, Germany

**Keywords:** Intermetallic compound, crystal structure, chemical bonding, reactivity, heterogeneous catalysis, ethylene epoxidation, ethylene oxide, 60 New topics / Others, 106 Metallic materials, 205 Catalyst / Photocatalyst / Photosynthesis, 301 Chemical syntheses / processing, 401 1st principle calculations, 503 TEM, STEM, SEM

## Abstract

The ethylene epoxidation is a challenging catalytic process, and development of active and selective catalyst requires profound understanding of its chemical behaviour under reaction conditions. The systematic study on intermetallic compounds in the Ca–Ag system under ethylene epoxidation conditions clearly shows that the character of the oxidation processes on the surface originates from the atomic interactions in the pristine compound. The Ag-rich compounds Ca_2_Ag_7_ and CaAg_2_ undergo oxidation towards *fcc* Ag and a complex Ca-based support, whereas equiatomic CaAg and the Ca-rich compounds Ca_5_Ag_3_ and Ca_3_Ag in bulk remain stable under harsh ethylene epoxidation conditions. For the latter presence of water vapour in the gas stream leads to noticeable corrosion. Combining the experimental results with the chemical bonding analysis and first-principles calculations, the relationships among the chemical nature of the compounds, their reactivity and catalytic performance towards epoxidation of ethylene are investigated.

## GRAPHICAL ABSTRACT

**Figure:**
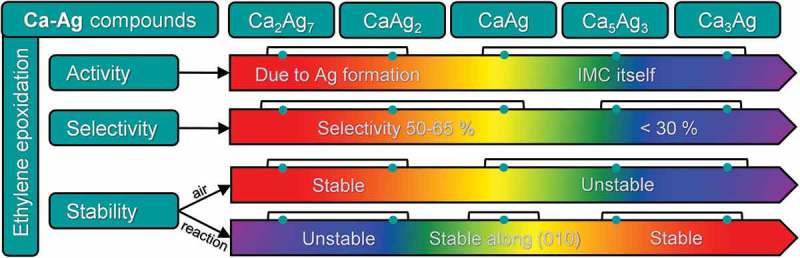

## 1. Introduction

Every industrial chemical process is an extremely complex and dynamic system in terms of physical, chemical and engineering parameters. In recent years, tremendous attention is focused on the changes of catalyst under dynamic reaction conditions [1]. Accordingly, the catalyst should be considered as a dynamic part of the catalytic system, and its completely different behaviour under operation as opposed to under steady-state conditions needs to be taken into account. These observations act as driving forces for development of advanced *in situ* and *operando* techniques [2–9], which give an insight into the crystal structure, surface morphology and composition of the catalyst.

Epoxidation of ethylene is an important chemical process from the industrial perspective and – simultaneously – a very complex catalytic reaction from the fundamental point of view [10,11]. This reaction belongs to the oxidation processes, and the presence of molecular oxygen in the gas stream *a priori* means possible surface reconstruction or even changes in the bulk of the catalyst material. Without knowing the micro- and crystal structure and the electronic state of the catalyst, it is difficult to define possible active sites or recognize potential reaction pathways, which are parts necessary for a reaction mechanism. The complexity of the material increases from the single crystal of elemental Ag through polycrystalline materials towards supported and highly promoted industrially used Ag/α-Al_2_O_3_ catalysts. Insertion of a second element either in the form of doping (e.g., Cu [9,12–14], Pd [15]) or alloys (Zn [16], Cd [17], Au [18]) leads to the preferential segregation to the surface and formation of oxidic phases due to the different reactivity of the components and random distribution of the elements in the crystal structure. Contrary to alloys, in (ordered) intermetallic compounds (IMCs) the atoms (preferentially) occupy certain crystallographic positions and chemical interactions can be properly described in terms of local chemical bonding. As a result, IMCs can be used either as stable and active catalysts themselves [19–21] or as precursors for the formation of efficient catalyst under reaction conditions. The latter variant can be realized in two modes: (i) via preparation of active catalysts using pre-treatment of precursor compounds (e.g. leaching, oxidation, etc.) [22–24], which is a known phenomenon dating back to 1920s with the discovery of Raney catalyst [25], or (ii) through formation of active catalyst as a result of the structural and compositional changes of the intermetallic compound under reaction conditions. There are numerous examples where intermetallic compounds were initially used as catalysts, but the actual catalytic performance had to be attributed to *in situ* formed metal particles supported on oxides [26,27], nitrides [28–30], hydrides [31], etc. To understand and predict the behaviour of an intermetallic compound under certain reaction conditions, knowledge of the chemical properties and reactivity is crucial, but such information remains scarce and hidden in the literature. The ordered crystal structures of IMCs open a possibility to use them as models for studying chemical reactivity [32,33].

In the context of ethylene epoxidation process, our previous studies clearly show that the reactivity of CaAg_2_ and CaAg to oxygen strongly depends on the crystal structure and chemical bonding features [34,35]. Here, a comprehensive overview of the reactivity of the binary compounds in the Ca–Ag system under ethylene epoxidation conditions is presented. The chemical changes of these Ca–Ag compounds were considered from different perspectives: (i) nature of the oxidant (not exclusively oxygen, but also carbon dioxide and water vapour), (ii) effect of the reaction temperature and (iii) addition of ethyl chloride as a promoter. The observed changes in the catalyst material under reaction conditions were linked to the catalytic performance. The experimental results, supported by chemical bonding analysis and first-principles calculations, strengthen experience on intermetallic compounds as catalyst precursors for the ethylene epoxidation and contribute to the fundamental understanding of their chemical properties.

## 2. Experimental

Single-phase samples of binary compounds were synthesized through the reaction of constituent elements (Ag shot, 1–6 mm in size, *Alfa Aesar*, 99.999%; Ca dendritic pieces, *Alfa Aesar*, 99.98%) at elevated temperatures. The components were weighed in corresponding ratios in a glove box and sealed under argon into tantalum containers. The latter were positioned into quartz tubes, which were sealed under vacuum. For initial reaction, samples were heated to 1000°C and dwelled at this temperature for 1 h, followed by cooling down to corresponding annealing temperature and holding for 240 h. The annealing temperatures were chosen based on the phase diagram [36] and own thermal analysis results: 670°C for Ca_2_Ag_7_, 570°C for CaAg_2_, 640°C for CaAg, 540°C for Ca_5_Ag_3_ and 430°C for Ca_3_Ag. After homogenization, the samples were quenched in ice water.

For sample characterization, the following bulk-sensitive techniques were applied: powder X-ray diffraction (PXRD), scanning electron microscopy (SEM), and differential thermal analysis combined with thermogravimetry (DTA/TG). PXRD patterns were recorded using Huber Imaging Plate Guinier Camera G670 (Huber Diffractionstechnik GmbH, Rimsting, Germany); Cu*K*α_1_ radiation (λ = 1.54059 Å) and internal standard LaB_6_ (*a* = 4.1569 Å). Ground powders were homogeneously distributed on vaseline-wetted kapton foil and fixed tightly between two such foils. The sample holders were taken out of Ar-filled box directly before the measurement. Phase analysis was performed by comparing collected patterns with the calculated ones based on the available crystallographic data (ICSD [37], CRYSTMET [38]) using WinXPow software [39]. For indexing of PXRD patterns and refinement of crystal structures, WinCSD software package [40] was applied.

Scanning electron microscopy (JEOL 7800F with an attached EDX/EBSD system: Quantax 400, Bruker, Silicon-Drift-Detector; JEOL GmbH, Freising, Germany) was employed for: (i) semi-quantitative estimation of composition via EDXS analysis and (ii) elemental mapping and morphology imaging of small pieces of as-synthesized and as-processed samples. Due to sample sensitivity to air and moisture, all preparation steps were carried out in an Ar-filled glove box, and a shuttle system was used for sample transfer into SEM. Specimens were cold fixed in silver containing epoxy resin or mounted on graphite tabs. Silicon carbide paper and diamond powder with 3 µm or smaller grain size were used for surface polishing.

Differential thermal analysis (Netzsch STA 449 F3 Jupiter; NETZSCH-Geraetebau GmbH, Germany) was accompanied by thermogravimetry and mass spectrometry (Pfeiffer OmniStar GSD 301 O3; Pfeiffer Vacuum GmbH, Germany). Two sets of conditions were applied: (i) ‘*zero conversion*’ mimicking conditions of ethylene epoxidation (O_2_: C_2_H_4_: He = 7: 35: 58 vol. %, total flow – 10 ml min^−1^) and (ii) ‘*full conversion*’ assuming that all oxygen is consumed to form CO_2_ and H_2_O (CO_2_: H_2_O: C_2_H_4_: He = 4.7: 4.7: 32.6: 58 vol. %, total flow – 40 ml min^−1^); Table S1.

Ethylene epoxidation experiments were performed using samples ground to two particle size fractions: 315–500 μm and 500–1000 μm. These powders were placed between two layers of inert corundum (Alodur WSK F60, 250–315 μm in size) in tubular stainless steel reactors (R0139, inner diameter 6 mm). After pre-heating to 160°C under nitrogen flow, further heating up to 230°C was accompanied with the switch to reaction gas mixture (35 vol. % C_2_H_4_, 7 vol. % O_2_, 5 vol. % Ar as internal standard and 53 vol. % N_2_ as carrier gas). All experiments were performed at ambient pressure, whereas temperature (*T*), gas hourly space velocity (GHSV) and amount of ethyl chloride (EC) promoter in the gas feed were varied (230–350°C, 1000–2000 h^–1^, 0–4.5 ppm, respectively). Gas chromatography (GC) and mass spectrometry (MS) were used for gas product analysis [41].

Two first-principles full-potential all-electron codes, namely the full-potential local orbital (FPLO) [42] and the Fritz-Haber Institute *ab initio* molecular simulations (FHI-aims) [43], both using atom-centred numerical orbitals were employed in the electronic structure calculations. The generalized gradient approximation (GGA) as parametrized by Perdew, Burke and Ernzerhof [44] was applied. The fully optimized bulk structures were used to generate surface models. Pristine surfaces and surfaces with adsorbed species were optimized by keeping the surface lattice vectors and the atoms of the bottom few layers fixed. The force criterion for atoms in both bulk and surface calculations was 5 meV Å^−1^. Surface energy and adsorption calculations were carried out within the slab approach by the FHI-aims package. The vacuum, separating the slabs, had a thickness of about 20 Å. The electric dipole corrections were included in the case of asymmetrical slabs [45,46]. The position-space chemical bonding analysis is based on the electron localizability approach with the electron localizability indicator (ELI) being computed in the ELI-D representation [47–49]. The electron density (ED) and ELI-D were calculated by the help of modules implemented in FPLO [50] and FHI-aims [51]. The program DGrid [52] was used for the topological analysis of the ED and ELI-D.

## 3. Binary Ca-Ag compounds: bulk properties

Before focusing on the chemical behaviour of binary Ca–Ag compounds under oxidative ethylene epoxidation conditions, a short overview of their bulk properties (crystal and electronic structure, nature of interatomic interactions) is presented.

### 3.1. Crystallographic features

**Figure 1.**
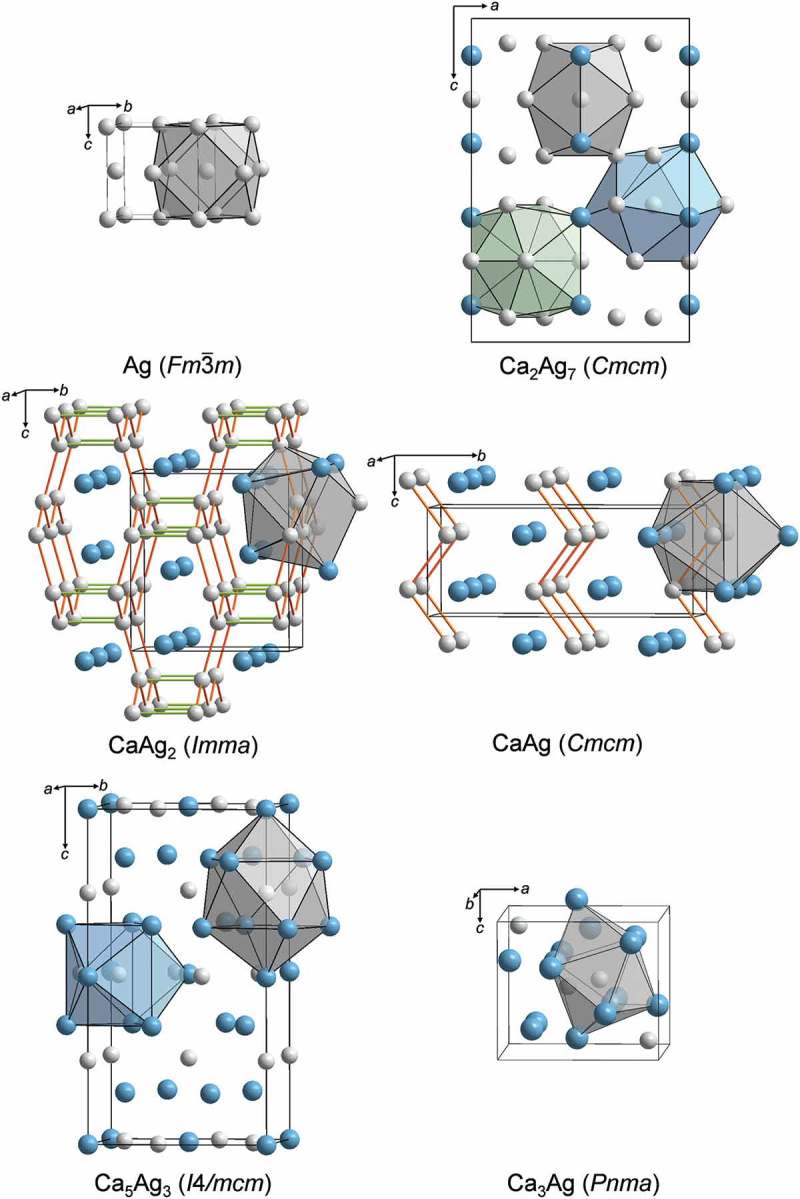
Crystal structures of elemental silver and Ca–Ag compounds with coordination polyhedra of Ag atoms (Ag *grey*; Ca *blue*).

The binary system Ca–Ag [36,53–55] is characterized by at least five compounds. They crystallize with unique crystal structures (Figure 1, Table S2), are formed by different reactions and have distinct physical properties. The crystal structure of the Ag-richest compound Ca_2_Ag_7_ (Yb_2_Ag_7_ type of crystal structure) [56,57] is closely related to that of elemental *fcc* Ag [58]: (i) three types of crystallographically distinct Ag atoms have distorted cubooctahedral atomic environment with coordination number CN = 12, (ii) the orthorhombic deformation of the unit cell leads to the formation of distorted hexagonal layers by Ag1 and Ag2, whereas Ag3 and Ca build trigonal layers, and (iii) the structure is formed via stacking of [Ag_4_Ca_2_], [Ag_6_] and [Ca_2_Ag_4_] slabs along [001]. Further dilution of Ag by Ca leads to the formation of CaAg_2_ (KHg_2_ type) [59] with a 3D framework of planar honeycomb nets of Ag atoms (Ag-Ag contacts in *orange*, Figure 1), linked via longer Ag-Ag contacts (in *green*, Figure 1) along [010]. Calcium atoms are located in the cavities of this atomic arrangement [34]. In the equiatomic compound CaAg (*α*-TlI type), the zig-zag chains of Ag atoms along [001] are separated by Ca atoms [35,59,60]. The silver atoms are surrounded by seven calcium and two silver atoms in the form of trigonal prisms with three additional vertices in front of side faces (CN = 9). The trigonal prismatic environment of Ag atoms is preserved for Ag2 atoms in the tetragonal Ca_5_Ag_3_ structure (Cr_5_B_3_ type) [61], whereas for Ag1 atoms the nearest neighbours form a tetragonal antiprism with two additional atoms (CN = 10). The crystal structure of the Ca-richest compound (Ca_3_Ag) was not reported in literature; only orthorhombic symmetry (space group *Cmcm*) was suggested from first-principles calculations [62]. Based on PXRD data, the crystal structure of Ca_3_Ag was determined and assigned to the Fe_3_C structure type (the detailed information is presented elsewhere). In this crystal structure, Ag atoms are at the centres of trigonal prisms with three additional atoms, all being Ca (CN = 9). It is clearly seen, that with increasing Ca content, the coordination number of Ag atoms decreases (Figure 1) from 12 (cuboctahedron in *fcc* Ag) to 9 (trigonal prism with three additional atoms in Ca_3_Ag case). Furthermore, the number of Ag-Ag contacts reduces from 12 in *fcc* Ag down to their complete absence in Ca_3_Ag (Table S2).

Among the Ca–Ag compounds mentioned in the literature, Ca_2_Ag_9_ and Ca_3_Ag_2_ were not obtained in this study. Composition Ca_2_Ag_9_ was assigned [53] for a compound which was previously reported as ‘Ag_4_Ca’ [63]. Its existence cannot be excluded due to the presence of tiny unindexed reflections in the PXRD patterns of Ca_2_Ag_7_, but due to the high ductility of the samples, the PXRD analysis as well as growth of single crystals are strongly hampered. A formation of an unknown phase at 59 at. % Ca (close to the composition Ca_3_Ag_2_) through a supposed peritectic reaction at 589°C was reported [53], but the formation temperature and composition of Ca_3_Ag_2_ are very close to those of Ca_5_Ag_3_, hindering the synthesis of Ca_3_Ag_2_ as single-phase sample.

### 3.2. Thermodynamic stability

The Ca–Ag binary system has been studied experimentally and computationally [36,53–55,62–66]. An assessment of the Ca–Ag phase diagram was made based on the first-principles calculations and experimental data [62]. The Gibbs energies of formation of four compounds in the Ca–Ag system were determined by potentiometric measurements at 830 K as Ca_2_Ag_9_: −15.7 kJ mol^−1^, Ca_2_Ag_7_: −18.9 kJ mol^−1^, CaAg_2_: −22.5 kJ mol^−1^ and CaAg: −25.0 kJ mol^−1^ [64]. These values are in good agreement with the results of thermodynamic modelling by the CALPHAD approach [65] and with the standard formation enthalpies measured by the solution calorimetric method: −18.8 kJ mol^−1^ for Ca_2_Ag_7_, −24.1 kJ mol^−1^ for CaAg_2_, −28.0 kJ mol^−1^ for CaAg [66].

**Figure 2.**
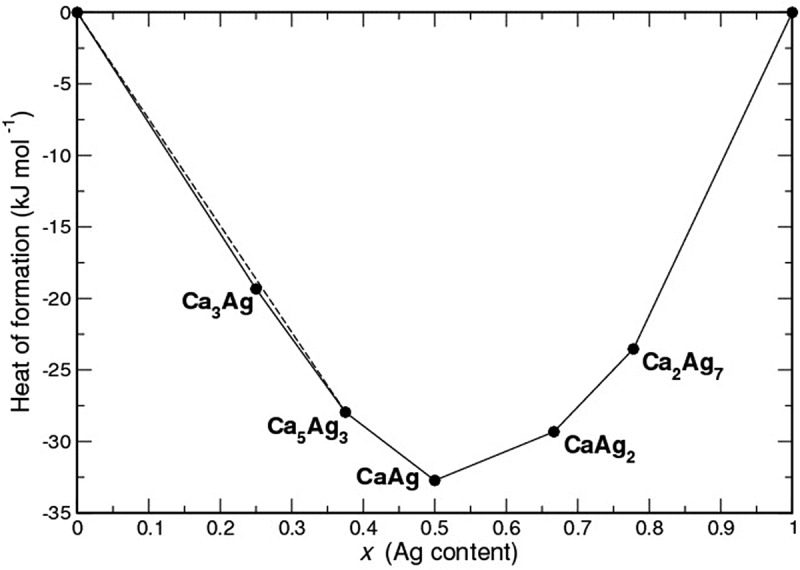
Calculated composition-dependent heats of formation for the compounds in the binary system Ca–Ag.

The crystal structures of Ca–Ag binary compounds used in the present study were optimized at the GGA level. According to the formation energies calculated from the electronic total energies, all five compounds are on the tie-line of the convex hull and therefore thermodynamically stable (Figure 2). Calculated values (−23.54 kJ mol^−1^ for Ca_2_Ag_7_, −29.32 kJ mol^−1^ for CaAg_2_, −32.73 kJ mol^−1^ for CaAg, −27.95 kJ mol^−1^ for Ca_5_Ag_3_ and −19.34 kJ mol^−1^ for Ca_3_Ag) agree well with the previous results [62], which include phonon contributions to the Helmholtz free energy (−22.87, −28.63, −32.74, −28.16 kJ mol^−1^ at room temperature for Ca_2_ Ag_7_, CaAg_2_, CaAg and Ca_5_Ag_3_, respectively). In all these studies the lowest formation energy is observed for the equiatomic CaAg.

### 3.3. Tendencies in electronic structures

**Figure 3.**
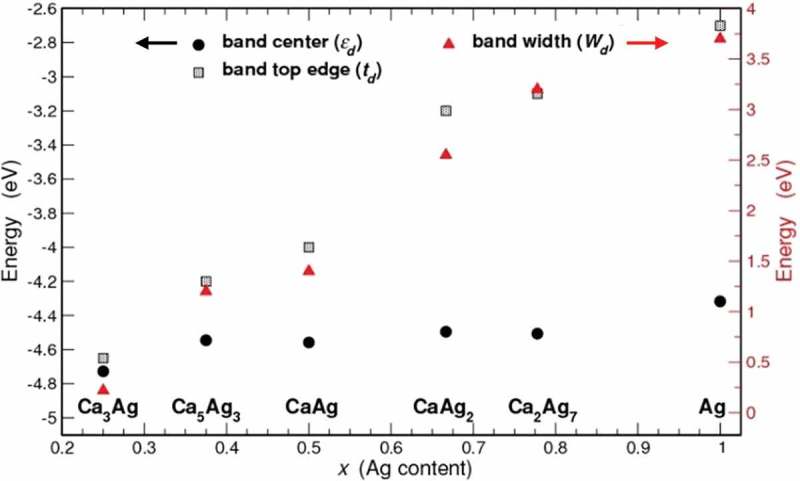
Band centre, band top edge and band width of Ag *4d* states versus Ag content in Ca–Ag binary compounds.

The calculated electronic total and partial densities of states (DOS) for five Ca–Ag compounds are shown in the *Supporting Information* (Figure S1). The changes in DOS moving from the Ag-richest Ca_2_Ag_7_ to the Ag-poorest Ca_3_Ag reflect mainly the changes in Ag *d*-Ag *d* hybridization, which depend on the number of Ag-Ag contacts. The *d* band width (*W_d_*) decreases with decreasing near neighbour numbers, and as the nature of *d* states start to become more localized (atomic) the whole *d* band moves further away from the Fermi energy (*E_F_*). This downward shift can be quantified by the distance of the *d* band maximum to the *E_F_* (top edge *t_d_*) and the *d* band centre (*ε_d_*). Figure 3 shows the variations of *W_d_, t_d_* and *ε_d_* as a function of Ag content. The *fcc* Ag, where each Ag atom has 12 neighbours at a distance of 2.892 Å (Table S2), has the widest band (3.7 eV) and its band top edge lies highest at −2.7 eV, whereas in Ca_3_Ag homonuclear CN of silver atoms becomes zero and correspondingly *W_d_* is reduced to 0.2 eV. The *d* band centres, however, vary in a narrow range of −4.3 to −4.7 eV.

### 3.4. Chemical bonding analysis

**Figure 4.**
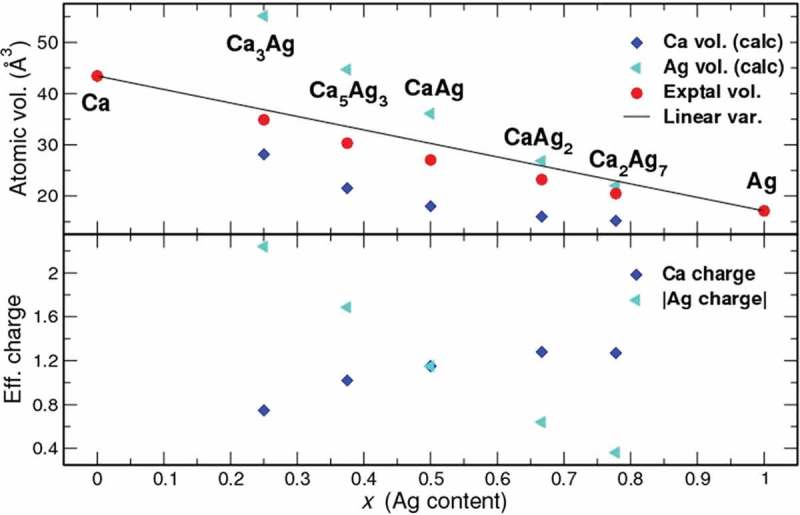
QTAIM volumes (top) and effective charges (bottom) of Ca and Ag atoms in different Ca–Ag binary compounds.

The experimental average volumes per atom in the elemental structures and the Ca–Ag compounds are displayed in Figure 4. The solid line joining *fcc* Ca and *fcc* Ag shows the linear behaviour expected from Vegard’s rule. However, the values for the Ca–Ag compounds (*red full circles*, Figure 4) indicate a negative deviation (smaller volumes). This implies that the nature of chemical bonding in the compounds is different from that in the elemental solids [67,68]. One obvious feature originating from the electronegativity difference between Ca and Ag is the charge transfer from Ca to Ag (Figure 4). The average effective QTAIM charge of Ca increases as Ag content increases and reaches a maximum value of about +1.3, characteristic for compounds with high bond polarity: +1.20 in Ca_2_SiIr_2_ [69], +[1.3,1.5] in CaSi_3_ [70], +[1.28,1.42] in Ca_12_[Mn_19_N_23_] [71]. The average volume of the Ca species decreases and reaches asymptotically a minimum value of 15 Å^3^/atom. With increasing Ag content the negative charge of silver species decreases and the average QTAIM volume of Ag atoms decreases at the same time reflecting the decreased number of electrons contained in Ag QTAIM basins.

**Figure 5.**
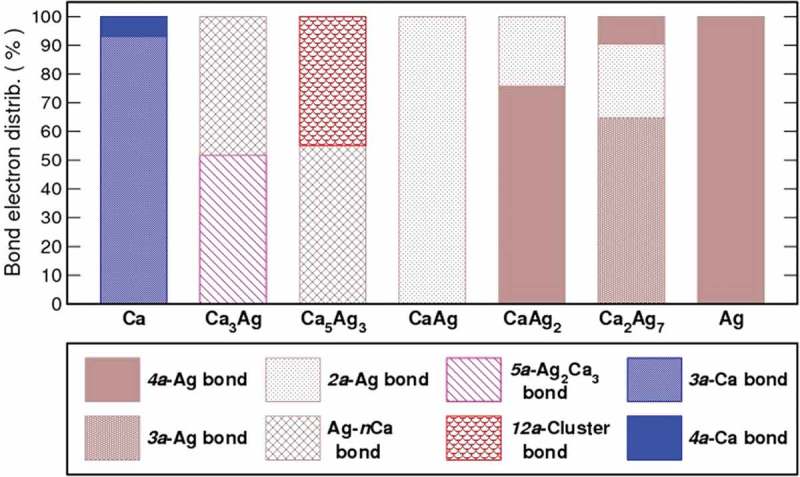
Distribution of bond electrons over different bond types for each Ca–Ag compound. The bond types are described in the text.

The electron localizability approach enables analysis of the chemical bonding in position space. The compound-specific results of such analysis are presented in the Supporting Information (Figures S2-4). The following bond types are found. In the (four-atomic [72]) *4a*-Ag_4_ bond all or most of the electrons in the bond basins are contributed by four Ag atoms. The individual Ca contributions to such bonds are less than 5%. Similarly, the *3a*-Ag_3_ and *2a*-Ag-Ag bonds are defined. The Ag-*n*Ca interaction involve one Ag and two or three Ca atoms. The Ag atom provides most of the bond electrons with individual Ca atoms contributing between 5% and 10%. The polarity of this bond type is high. The *5a*-Ag_2_Ca_3_ bond is formed by two Ag and three Ca atoms all having comparable contributions, which reduces its polar character. The cluster bond [73] with the participation of eight Ag and four Ca atoms (12 atomic) has a very large bond electron population of 6.35 electrons. The polarity of this bond is further reduced with respect to previous ones. The last two types are Ca-only bonds (*3a*-Ca_3_ and *4a*-Ca_4_) found in *fcc* Ca. For each bond type, the total number of bond electrons is obtained and its ratio to the compound’s total number of bond electrons (i.e., the ELI-based valence region electron count) is calculated (Figure 5). The Ag-rich compounds are characterized by multi-atomic Ag-dominated polar covalent bonds, whereas the Ca-rich ones reveal multi-atomic bonds with comparable Ag and Ca contributions. The equiatomic CaAg with its *2a*-Ag-Ag bonds is in-between variant of these two different bonding scenarios.

### 3.5. Surface energy calculations

**Figure 6.**
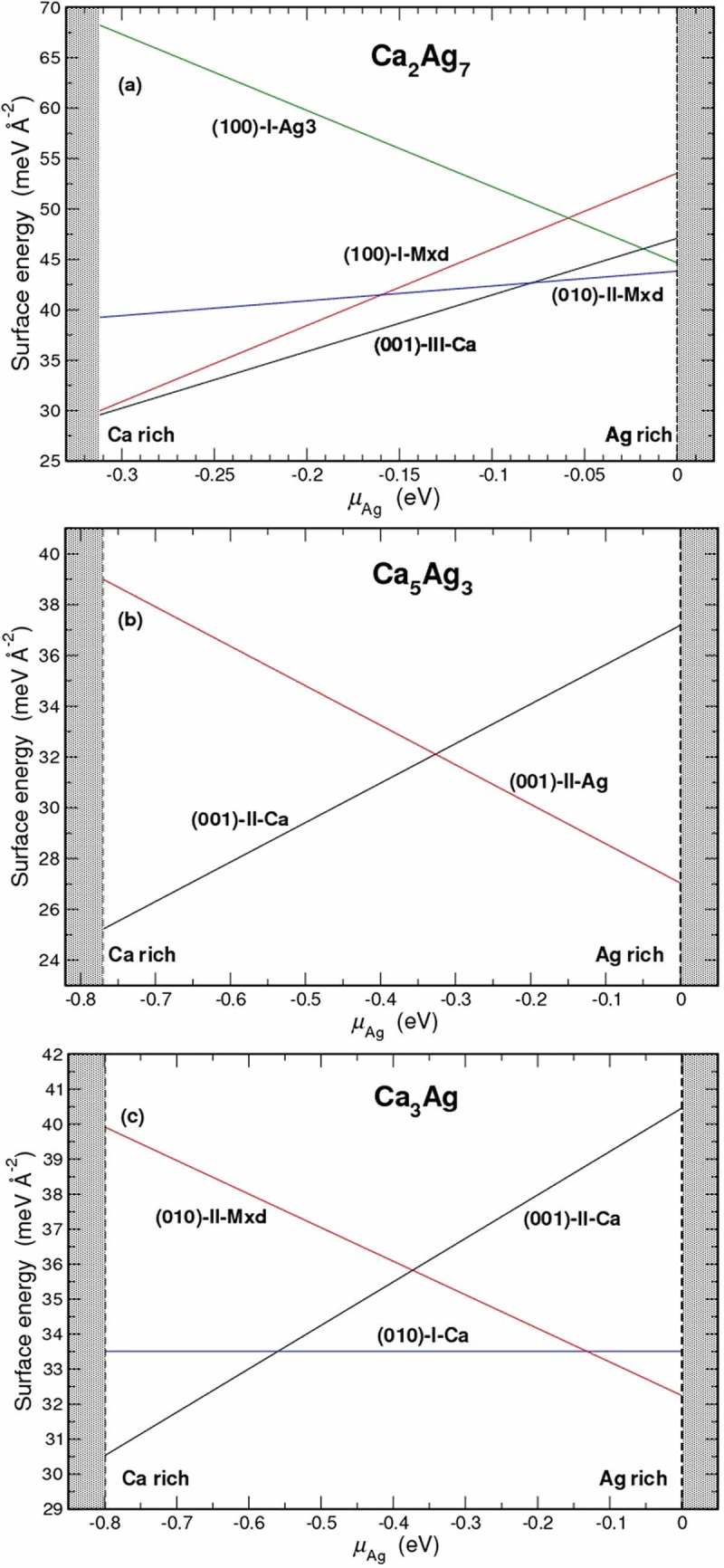
The most competitive surface terminations obtained for Ca_2_Ag_7_ (a), Ca_5_Ag_3_ (b) and Ca_3_Ag (c).

Surface energies of the pristine surfaces (100), (010) and (001) were calculated using the slab geometry. Since a slab has two surfaces, it is necessary to have identical surfaces on both sides. In most cases, this requires nonstoichiometric slabs, and the chemical potentials for Ca (*µ*_Ca_) and Ag (*µ*_Ag_) have to be introduced in order to account for the added or removed atoms with respect to the bulk composition. In Figure S5 the surface energies as a function of the chemical potential of Ag are presented. Apart from CaAg, all Ca–Ag binaries have a Ca-terminated surface as the preferred surface in Ca-rich environments (covering a wider range of *µ*_Ag_ values) and either Ag- or mixed-terminated surfaces in Ag-rich environments. The surfaces with the lowest surface energies calculated for Ca_2_Ag_7_, Ca_5_Ag_3_ and Ca_3_Ag are shown in Figure 6, whereas such information for CaAg_2_ and CaAg compounds was presented elsewhere [34,35].

In terms of cleavage energies, the Ag-rich compounds behave differently from others (Table 1). Two different cleavage planes each for the Ca_2_Ag_7_ (001) and (010) surfaces have comparable cleavage energies (within 0.5 meV Å^−2^). In CaAg_2_ the best surfaces along the principal directions [100], [010] and [001] have very close cleavage energies [34]. On the other hand, in CaAg, Ca_5_Ag_3_, and Ca_3_Ag there are clearly preferred cleavage planes implying anisotropic behaviour for these compounds. In particular, the favoured surface of CaAg is a Ca-terminated (010) surface over the full range of *µ*_Ag_ [35].

**Table 1.** Cleavage energies (in meV Å^−2^) computed for Ca–Ag binary compounds*.

Ca_2_Ag_7_	CaAg_2_ [34]	CaAg [35]	Ca_5_Ag_3_	Ca_3_Ag
(001)-I: 46.48	(100)-I: 49.00	(010)-III: 32.00	(001)-II: 32.11	(010)-I: 33.51
(001)-III: 46.58	(010)-I: 49.80	(111): 37.50	(100)-I: 38.40	(100)-I: 36.45
(010)-I: 46.60	(001)-I: 49.70	(101): 38.70		(001)-I: 36.52
(010)-II: 47.00				(010)-II: 36.78

*The cleavage planes are denoted according to Figures S6–S8.

## 4. Characterization of synthesized compounds

The assessment of intermetallic compounds as catalysts requires the careful characterization of the as-synthesized material to relate the catalytic performance to either the chemical nature of IMCs or the changes occurring under reactive atmosphere. The as-prepared Ca–Ag samples were handled under inert atmosphere, preventing their possible oxidation by exposure to air. The Ag-richest compound Ca_2_Ag_7_ is extremely ductile (similar to elemental Ag), whereas CaAg_2_ and CaAg are brittle. The PXRD data collection for Ca_2_Ag_7_ is only possible after mechanical filing of the sample and subsequent annealing of the powder. With Ca content larger than 50 at. %, the samples (e.g., Ca_5_Ag_3_ and Ca_3_Ag) are soft. The mechanical properties influence the quality of the collected X-ray powder diffraction data (Figure S9). The synthesis procedure described in the *Experimental* part allows the preparation of single-phase binary compounds. Only a few unidentified broad reflections, pointing out the low crystallinity of the admixture phase, were observed in the PXRD pattern of the Ca_2_Ag_7_ sample (Figure S9). These reflections may correspond to the Ca_2_Ag_9_ phase, whose crystal structure is still unknown. The synthesis of Ca_5_Ag_3_ resulted in the presence of the target phase and some Ca_3_Ag_2_, which is concentration- and temperature-wise very close to Ca_5_Ag_3_. The sensitivity of the Ca–Ag samples to air was monitored via PXRD measurements: successive PXRD patterns (six measurements, each 20 min, summed up for better statistics) do not show any changes for Ca_2_Ag_7_ and CaAg_2_. For the equiatomic CaAg additional broad reflections appear already at the second recording and their contribution increases with measurement time. The formation of additional amorphous-like oxidation products is even more pronounced for Ca_5_Ag_3_ and Ca_3_Ag.

## 5. Ca–Ag compounds under ethylene epoxidation conditions

The catalytic performance of intermetallic Ca–Ag compounds towards production of ethylene oxide was investigated by varying different experimental conditions: (i) temperature (*T*), (ii) gas hourly space velocity (GHSV), and (iii) concentration of ethyl chloride (EC) promoter (Figure S10). The main measures for efficiency are selectivity towards ethylene oxide (*S*_EO_) and conversion of ethylene (*C*_C2H4_); the obtained values are summarized in Table 2.

**Table 2.** Catalytic performance of Ca–Ag compounds for ethylene epoxidation: selectivity towards ethylene oxide (*S*_EO_, %) and conversion of ethylene (*C*_C2H4_, %) as response to varied experimental conditions (temperature (*T*, °C), gas hourly space velocity (GHSV, h^−1^) and concentration of ethyl chloride promoter (EC, ppm)).

				*T* increase***: 250 → 280 °C		*T* decrease***: 250 → 200 °C		GHSV decrease***: 2000 → 1000 h^−1^		EC decrease***: 2.5 → 0 ppm	
Compound	*t*_ind_*, h	*S*_EO_**, %	*C*_C2H4_, %	*S*_EO_, %	*C*_C2H4_, %	*S*_EO_, %	*C*_C2H4_, %	*S*_EO_, %	*C*_C2H4_, %	*S*_EO_, %	*C*_C2H4_, %
Ca_2_Ag_7_	250	56–58	1.5		↑4	↓ 40	↓ <1		↑ 3	↓ 28	↑ 3
CaAg_2_	30	64	3	↓ 52	↑6	↓ 58	↓ <1	↑ 68	↑ 4	↓ 36	↑ 4
CaAg	100	35	<1			↓ 6		↑ 39	…	↓ 22	
Ca_5_Ag_3_	…	8	<1	↑ 12		↓ 3		↑ 12	…		
Ca_3_Ag	…	4	<1	…	…	…	…	↑ 8	…	…	…

* Induction time (*t*_ind_, h) represents the time for catalytic activity to reach the quasi steady state.

** Selectivity values are given at corresponding level of ethylene conversion; furthermore, these values represent the values of selectivity at standard temperature of 250°C.

*** The response of selectivity and conversion to temperature, GHSV and EC concentration changes are presented: arrow up (down) corresponds to increase (decrease) of selectivity or conversion; the numbers represent the values, reached at final *T*, GHSV or EC concentration; empty cells mean unchanged catalytic performance compared to standard conditions.

### 5.1. Duration of the induction phase

The oxidative conditions of ethylene epoxidation process require time *t*_ind_ for the catalytic activity to reach the quasi steady state. This time frame is referred to as the induction phase. The attainment of the steady state can be inferred by noting when the monotonic changes in catalytic measures reach stable values. For Ag catalysts, these changes mainly correspond to the formation of surface oxide layer and/or growth of bulk oxide depending on the reaction conditions [74]. In case of the doped or alloyed Ag catalysts, e.g. the Ag-Cu, Ag-Cd, Ag-Pd, Ag-Zn systems, the picture becomes even more complicated because of possible segregation of one of the components to the surface and/or bulk transformation of the material [9,14–17].

**Figure 7.**
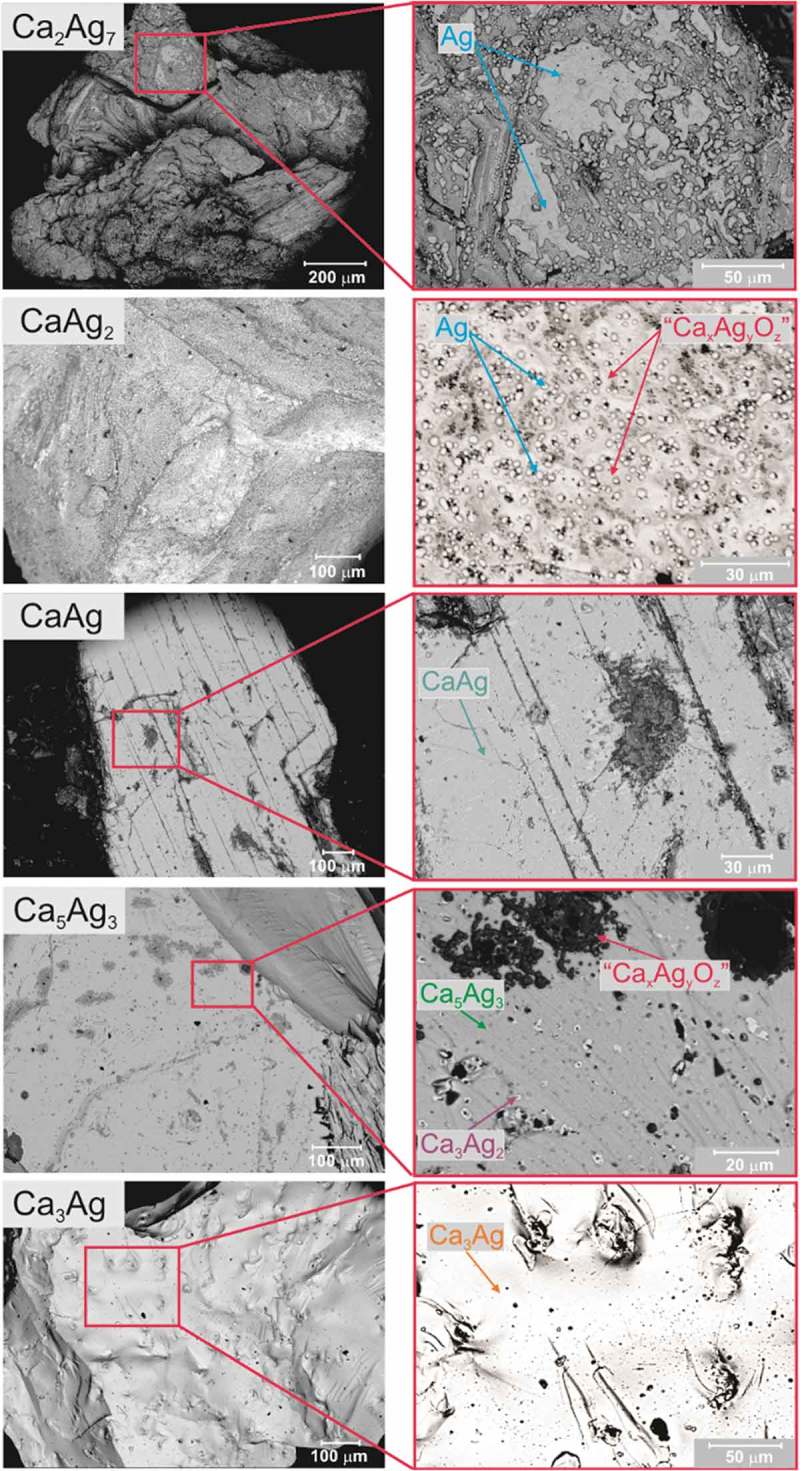
Particle shape and surface morphology of the Ca–Ag compounds after ethylene epoxidation (BSE images, material contrast, 15 kV): general view of particles (*left panel*) and magnification of selected areas (*right panel*). The identified phases are marked on the *right panel* figures. In case of CaAg, oxidation happens only on mechanically damaged areas (for details, see [35]).

The induction phase for almost all Ca–Ag compounds can be identified by monitoring the selectivity towards ethylene oxide. In the case of Ca_2_Ag_7_, the selectivity reaches the stable values of 56–58% at conversion around 1.5% only after 250 h under stream, whereas CaAg_2_ has a significantly shorter induction phase (*t*_ind_ = 30 h and *S*_EO_ = 64% at *C*_C2H4_ = 3%). It was shown previously that CaAg_2_ undergoes oxidation towards elemental Ag and a complex CaO/Ca(OH)_2_/CaCO_3_ support due to the isotropic chemical bonding with no evidence of preferential cleavage or formation of any protective layer [34]. In this case, oxidation is mostly driven by thermodynamics, since the reaction enthalpy of CaAg_2_ + ½ O_2_ → 2Ag + CaO is −252.8 kJ mol^−1^. In these terms, Ca_2_Ag_7_ behaves similarly to CaAg_2_ (Ca_2_Ag_7_ + O_2_ → 7Ag + 2CaO, Δ*H* = −104.2 kJ mol^−1^) and additional reflections corresponding to *fcc* Ag were detected in the PXRD patterns after catalysis. The formation of elemental silver was additionally evidenced via scanning electron microscopy, including EDXS analysis (Figure 7). Contrary to CaAg_2_, silver particles formed during the oxidation of Ca_2_Ag_7_ tend to aggregate, thus forming Ag ‘islands’. Most likely, the significantly slower oxidation rate of Ca_2_Ag_7_ compared to CaAg_2_ (estimated by comparing *t*_ind_), allows for Ag aggregation and leads to the sintering of the Ag particles. The formation of large Ag areas, which cover the non-oxidized part of Ca_2_Ag_7_, hinders the access of gas reactants (particularly O_2_) for further bulk oxidation of Ca_2_Ag_7_. The remaining core of Ca_2_Ag_7_ particles after exposure to ethylene epoxidation conditions was evidenced via PXRD and SEM. The sintering of the Ag particles is most probably also the reason for the reduced catalytic activity compared to *in situ* formed Ag from the CaAg_2_ precursor. It is well known that the particle sintering is the main deactivation mechanism of Ag-based catalysts with time on stream [75–78].

In case of CaAg, selectivities change within the first 100 h and reach maximum values of *S*_EO_ = 35% at *C*_C2H4_ = 0.5%, which are relatively low compared to the Ag-richer compounds. Contrary to CaAg_2_, CaAg remains stable in bulk under ethylene epoxidation conditions despite its oxidation, CaAg + ½O_2_ → Ag + CaO, is thermodynamically favourable with Δ*H* = −508.5 kJ mol^−1^. The stability of CaAg is due to the formation of a stable and ordered Ca-O passivation layer on the (010) surface, which is also the preferred cleavage surface [35]. The oxidation takes place only on stacking faults and mechanically damaged regions (Figure 7). The presence of the Ca-O passivating layer is most probably also the reason for the significantly diminished catalytic performance.

The induction phase for the Ca-rich compounds Ca_5_Ag_3_ and Ca_3_Ag is not very pronounced due to the very poor performance of these catalysts towards formation of ethylene oxide (*S*_EO_ = 8% at *C*_C2H4_ =1% for Ca_5_Ag_3_ and basically non-detectable for Ca_3_Ag). The comparison of PXRD patterns taken before and after the catalytic experiments does not reveal any difference. More careful microscopic studies on Ca_5_Ag_3_ samples after catalysis clearly show randomly distributed regions, whose compositions are enriched in Ca, Cl and O with accompanying depletion of Ag (*black* volcano-like areas in Figure 7). There are also plenty of homogeneously distributed regions, composed of cores enriched in calcium and oxygen according to EDXS analysis (*black* in Figure 7) and shells of Ag-richer phases (Ca_3_Ag_2_ according to EDXS analysis, *white* in Figure 7). These regions correspond to the first step of Ca_5_Ag_3_ oxidation. The SEM characterization of Ca_3_Ag (Figure 7) does not show any changes of the as-synthesized material and is in agreement with PXRD data.

### 5.2. Influence of temperature

The standard epoxidation experiments were performed at 250°C, but variation of the temperature (*T*) was carried out to specify the response of the catalytic properties to this factor (Table 2). With increasing temperature up to 280°C, the conversion of ethylene increases only slightly in case of Ca_2_Ag_7_, from 3% at 250°C up to 6% for CaAg_2_ and remains almost unchanged for the others.

The effect on the selectivity of Ca_2_Ag_7_ is unclear because the *T* increase step overlaps with the induction period. In the case of CaAg_2_ selectivity drops significantly down to 52%. The increase of the conversion rates and simultaneous decrease of the selectivity at elevated reaction temperatures are inherent features of elemental Ag catalysts [79,80], confirming the formation of elemental Ag upon oxidation of Ca_2_Ag_7_ and CaAg_2_ and its contribution to the catalytic activity. Increasing the temperature does not affect the performance of CaAg and that differentiates it from the Ag-richer compounds. A slight increase of selectivity (up to 12%) is observed for Ca_5_Ag_3_, however, error bars in selectivity estimation are large due to very low conversion rates.

At a reduced temperature of 200°C selectivity drops significantly in all cases, whereas ethylene conversion rate decreases dramatically for Ag-rich compounds (Ca_2_Ag_7_ and CaAg_2_) and remains almost unchanged and low for CaAg, Ca_5_Ag_3_ and Ca_3_Ag.

### 5.3. Influence of contact time and particle size distribution (PSD)

To enhance ethylene conversion, lower values of the gas hourly space velocity (GHSV) were applied and the samples with smaller particle size were used. The impact of these factors was clear only for Ag-rich compounds (Ca_2_Ag_7_ and CaAg_2_), where the conversion rates were slightly enhanced. Longer contact time of gas reactants with the catalyst and enlarged surface area exposed to the gas stream increased the oxidation rate of these compounds. For other Ca–Ag compounds, no significant enhancement of ethylene conversion was observed.

### 5.4. Promotion by ethyl chloride

The use of chlorinated hydrocarbons as promoters for ethylene epoxidation is widely applied on the industrial scale and allows to enhance the selectivity towards the desired ethylene oxide [11,81,82]. The mainstream opinion is that the combination of electronic and structural effects of chlorine allows to tailor the balance between different oxygen species (electrophilic and nucleophilic in nature) and, correspondingly, the reaction rates of different reaction pathways [83–87]. Depending on the coverage, chlorine can: (i) adsorb on surface or penetrate into subsurface at low coverages or (ii) completely poison (via blockage of oxygen chemisorption) the catalyst in case of high-concentration co-feeding [81,86,88–90]. The scenario of formation of highly mobile AgCl species leading to the restructuring and re-dispersion of Ag particles was also considered in the literature [91]. Interestingly, the replacement of the alumina support (industrially used) by CuO leads to CuO acting as a sponge for low concentrations of chlorine and is accompanied with formation of CuO/Cl derivatives [92].

For Ca–Ag compounds, the response of the catalytic performance on the presence of ethyl chloride (EC) in the gas stream is very pronounced. In the absence of EC, the selectivity drops drastically (more than twice) in the case of Ag-rich compounds (Ca_2_Ag_7_ and CaAg_2_) and this finding provides additional support for two claims: (i) the behaviour of these two compounds under ethylene epoxidation conditions is similar, and (ii) their catalytic response is due to the *in situ* formed Ag particles on a complex Ca-based support. CaAg is not so sensitive to the amount of EC in the stream, whereas for Ca_5_Ag_3_ and Ca_3_Ag its influence is difficult to estimate based solely on catalytic properties (Table 2).

The careful characterization of Ag-rich compounds as well as CaAg by bulk-sensitive techniques does not show any sign of reaction products with EC. Most probably, the catalytic response of these compounds is mainly governed by the geometrical and electronic structure changes of the *in situ* formed Ag particles upon EC co-feeding.

**Figure 8.**
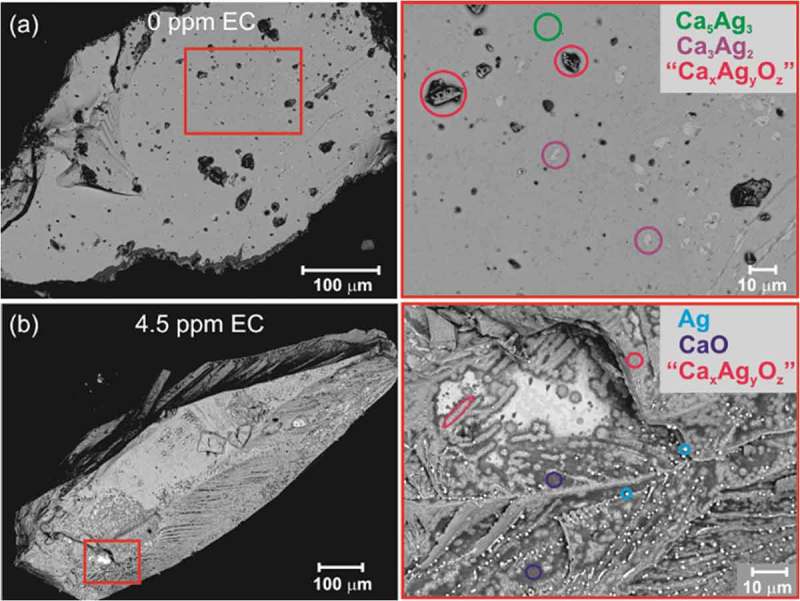
Morphology of Ca_5_Ag_3_ particles after ethylene epoxidation: without EC promoter (a) and with excess of it (b). The overview of particles (*left panels*) and enlargement on characteristic regions (*right panels*) are shown. The phases identified via EDXS analysis are marked in colours.

The inability to judge the impact of EC on the chemical properties of Ca_5_Ag_3_ and Ca_3_Ag at standard ethylene epoxidation conditions forced us to investigate their reactivity at elevated *T* (up to 350°C) without EC promoter and with excess of it (up to 4.5 ppm). The results of the experiment without EC promoter resemble those of the standard ethylene epoxidation test (2.5 ppm EC). The main feature is the appearance of surface areas enriched in calcium and oxygen with simultaneous depletion of Ag (dark areas, Figure 8). The amount of this product of partial oxidation of Ca_5_Ag_3_ (labelled ‘Ca*_x_*Ag*_y_*O*_z_*’ in the following) is larger compared to the sample after standard treatment, most probably due to the higher temperature. The morphology of the particles after the experiment with increased EC amount is completely different from those of other experiments. Almost the whole surface undergoes oxidation with formation of ‘Ca*_x_*Ag*_y_*O*_z_*’ intermediate (fluffy *dark grey* phase). Tiny nicely shaped particles of elemental Ag are located along the dendritic framework of ‘Ca*_x_*Ag*_y_*O*_z_*’. The undermost layer is mainly CaO. The formation of elemental silver as a result of Ca_5_Ag_3_ oxidation was observed only at elevated *T* and high-concentration EC co-feeding. No chlorine signal was detected in the microscopy studies, but its impact on the oxidation process is noticeable. The chemical behaviour of Ca_3_Ag under such conditions is similar to that observed for Ca_5_Ag_3_: (i) without EC the oxidation is even less pronounced, and (ii) with 4.5 ppm EC noticeable oxidation of Ca_3_Ag takes place with formation of a phase enriched in Ca, O and Cl and depleted in Ag (Figure S11).

The nucleophilic chlorine atom of the EC molecule tends to react with the cationic moieties of Ca in Ca_5_Ag_3_ and Ca_3_Ag, but the presence of oxygen in the stream makes the interaction of chlorine complicated due to the possible formation of Ca-Cl-O ternary intermediates, e.g. Ca_4_OCl_6_, Ca(ClO_2_)_2_ or Ca(ClO_4_)_2_ [93–97]. These types of compounds can act as oxidation agents for Ag^δ-^ oxidizing towards Ag^0^ and Ca^δ+^ to formal Ca^2+^ (in CaO). The reduced chlorine species are most probably volatile and may be removed by the gas stream. As a result, elemental silver, intermediate ‘Ca*_x_*Ag*_y_*O*_z_*’ and CaO are formed, which is in agreement with experimental observations (Figure 8). It should also be kept in mind that the amount of EC (even 4.5 ppm) is very small compared to the oxygen content in the gas stream (7 vol. %).

The reaction of EC with the surfaces of the Ca–Ag compounds was studied computationally, as an exemplary case, for the Ca-terminated (001) surface of Ca_5_Ag_3_. The 1 × 1 surface unit cell (~ 8 Å × 8 Å) was employed. Three different surface scenarios were considered: (i) adsorption of one EC molecule on the pristine surface, (ii) adsorption of one EC molecule on an oxygenated surface, and (iii) adsorption of one EC molecule and 12 oxygen atoms on the pristine surface. The first scenario is of interest to gain basic knowledge. Because the gas feed used in the experiments contains only ppm levels of EC but 7 vol. % O_2_, the second and third scenario are more relevant for the ethylene epoxidation conditions.

The outcome of the adsorption of EC on the pristine Ca-terminated (001) surface of Ca_5_Ag_3_ depends on the initial geometry of EC. When the molecular plane (the plane containing Cl, two C, and one of the H atoms) is parallel to the surface, and when it is perpendicular with the C–C axis still parallel to the surface with the Cl atom further away from the surface, the adsorption energies are about −0.26 eV. In these cases, the EC molecule remains intact. However, when the molecular plane is initially perpendicular to the surface (regardless of whether the C–C axis is parallel or perpendicular to it) with the Cl atom placed towards the surface, the Cl atom detaches from the molecule and forms bonds with three top surface Ca atoms. The adsorption energy for these cases is around −3.14 eV.

To investigate the adsorption of EC on an already oxygenated surface, the relaxed surface containing 11 oxygen atoms was taken as substrate, and one EC molecule was placed at different initial positions. The largest adsorption energies vary between −0.24 and −0.27 eV with the EC molecule intact and minimal changes in the substrate. This finding means that once the top layer Ca atoms are bound to the oxygen atoms, they show little affinity to the Cl atoms and adsorption process has no strong effect on the substrate and the molecule.

**Figure 9.**
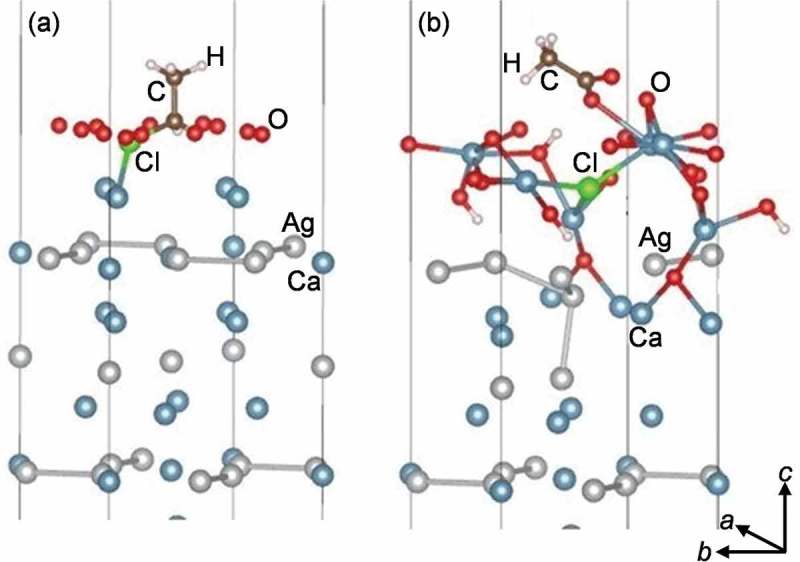
Initial (a) and relaxed (b) configurations of one EC molecule and 12 oxygen atoms above the pristine surface of Ca_5_Ag_3_. The length of the indicated Ca–Cl contact is 2.50 Å (a). The indicated Ca – Cl (Ca–O) contacts vary between 2.88 and 3.01 (2.22 and 2.68) Å (b).

The third scenario is simultaneous adsorption of the EC molecule and oxygen on the pristine surface. This possibility is simulated by considering one EC molecule and 12 oxygen atoms above the pristine surface (Figure 9(a)). In this case, both Cl and O atoms attack the Ca atoms disrupting the top two layers. The Cl atom penetrates beneath the top surface forming bonds with three Ca atoms, some oxygen atoms reach quite deep levels as in the oxygen-only calculations. Two hydrogen atoms detach from the EC and form OH groups. The C atom that has lost its Cl and H bonds attracts two O atoms into its vicinity. Additionally, there are various Ca–O bonds formed. However, the subsurface Ag atoms are less affected, and they remain below the top layer of Ca, Cl, O and H atoms (Figure 9(b)).

## 6. Water as oxidant

**Figure 10.**
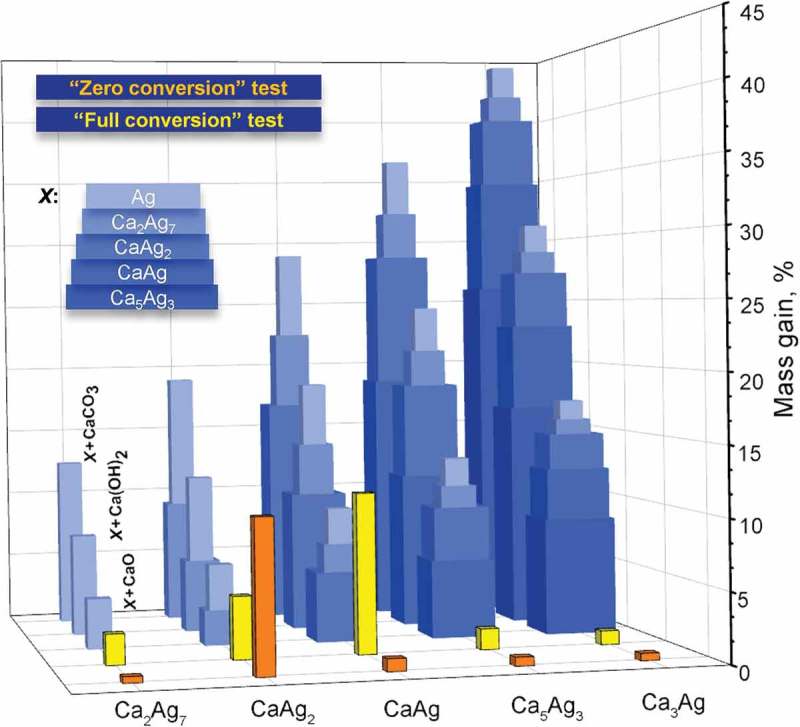
Mass gains as a result of the oxidation of Ca–Ag compounds: (i) calculated according to the scheme Ca*_x_*Ag*_y_* + O_2_ → *X* + CaO, where *X* = Ag, Ca_2_Ag_7_, CaAg_2_, CaAg and Ca_5_Ag_3_ (*blue bars*) and (ii) experimentally obtained during ‘*zero conversion*’ (*orange* bars) and ‘*full conversion*’ (*yellow* bars) tests. The presence of H_2_O vapour and CO_2_ in gas stream and possibility of Ca(OH)_2_ and CaCO_3_ formation was also considered.

The divergent behaviour of Ca–Ag compounds under exposure to air and under ethylene epoxidation conditions provoked thinking about other components of the system, which can act as oxidants, namely water vapour and carbon dioxide (undesired products of the combustion of ethylene). To address this issue, systematic DTA/TG-MS experiments were carried out in two modes: ‘*zero conversion*’ (the gas mixture is identical to the ethylene epoxidation test) and ‘*full conversion*’ (assuming that all available oxygen was used for ethylene oxidation towards CO_2_ and H_2_O). The experimental details are listed in Table S1. The oxidation of the Ca–Ag compounds was estimated based on mass gains during the experiments (Figure 10). Afterwards, all samples were characterized by PXRD and these results are summarized in Table 3.

**Table 3.** Results of DTA/TG-MS experiments on Ca–Ag samples: mass gains (%) as a result of material changes under different oxidative conditions accompanied via the phase analysis using powder X-ray diffraction.

	‘*Zero conversion*’		‘*Full conversion*’	
Compound	Mass gain, %	Phases*	Mass gain, %	Phases*
Ca_2_Ag_7_	0.45	Ca_2_Ag_7_+ Ag	2.20	Ag+Ca_2_Ag_7_**
CaAg_2_	10.8***	Ag+Ca_2_Ag_7_**+CaO**	4.50	Ag+Ca_2_Ag_7_+ CaO?
CaAg	0.90	CaAg+CaAg_2_	10.2/11.5	Ag+CaAg_2_+ CaAg+Ca_2_Ag_7_**+CaO+Ca(OH)_2_?
Ca_5_Ag_3_	0.63	Ca_5_Ag_3_+ CaAg	1.5/2.1	Ca_5_Ag_3_+ CaAg+CaAg_2_+?**
Ca_3_Ag	0.52	Ca_3_Ag+Ca_5_Ag_3_	1.0	Ca_3_Ag+Ca_5_Ag_3_+?**

*according to PXRD data﻿; **only traces are detected ; ***ethylene oxide, CO_2_ and H_2_O were detected from MS output.

Under reaction conditions (‘*zero conversion*’ mode), CaAg_2_ is the most unstable compound among Ca–Ag compounds and this is in agreement with experimental data (SEM, Figure 7) and the computational study of the surface which indicated the spontaneous segregation of elemental silver in O_2_-enriched atmosphere [34]. Exclusively in the case of CaAg_2_, the analysis of mass/charge curves shows the presence of ethylene oxide as well as carbon dioxide and water. Mass gain for Ca_2_Ag_7_ is small but continuously increasing, which can be explained by the higher availability of Ag atoms, which are segregating to the surface and thereby hindering access of oxygen to the Ca atoms. This explanation is also supported by the relatively long induction phase observed for Ca_2_Ag_7_ during the catalytic experiments (Table 2). CaAg and Ca-rich compounds (Ca_5_Ag_3_ and Ca_3_Ag) undergo oxidation only to a limited extent with formation of mainly neighbouring Ag-richer phases and CaO.

The ‘*full conversion*’ experiments clearly show the crucial effect of water on the stability of the Ca–Ag compounds. In general, the oxidation of all Ca–Ag compounds is more pronounced in the presence of H_2_O in the stream (Figure 10). Only the oxidation of CaAg_2_ is more obvious and quicker in O_2_-enriched atmosphere, whereas the presence of exclusively water vapour led to continuous and relatively slow oxidation of CaAg_2_ with formation of the intermediate product Ca_2_Ag_7_ and, finally, elemental Ag.

The prominent example for the impact of water is the chemical behaviour of CaAg, which remains relatively stable in the presence of O_2_, whereas the presence of H_2_O in the gas stream led to immediate oxidation with significant mass gain (Table 3, Figure 10). As mentioned above, its stability in the presence of O_2_ in the stream is explained by the formation of a very stable Ca-O surface structure, which acts as a passivating layer protecting bulk against further corrosion [35].

**Figure 11.**
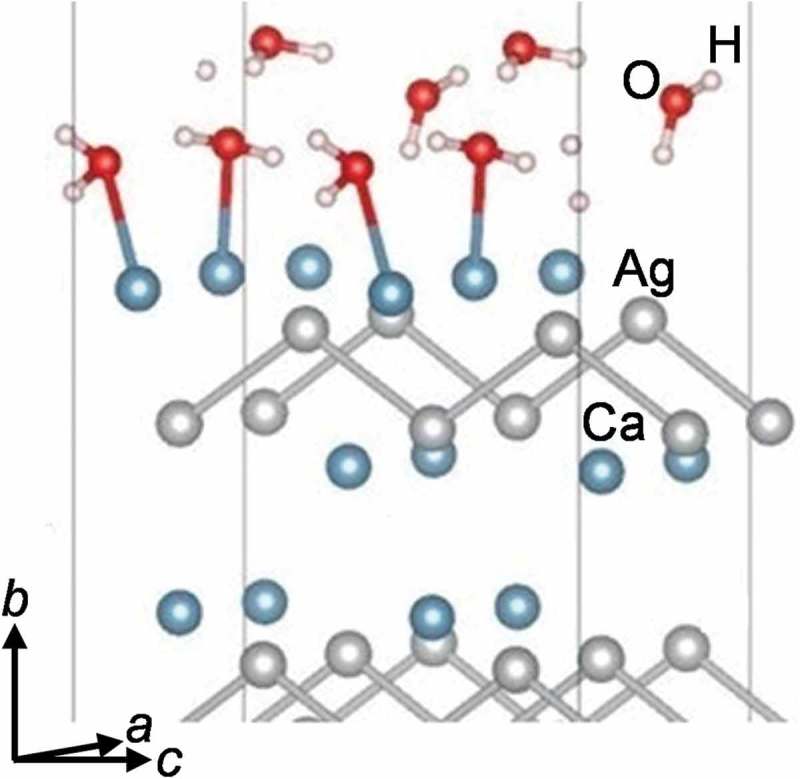
The relaxed arrangement of 8 water molecules on the pristine 2 × 2 CaAg (010) surface. There are four Ca atoms in the surface unit cell. The indicated Ca–O distances are 2.47–2.48 Å. The remaining four water molecules are too far from the surface to react.

To shed light on the impact of water on the oxidation process of CaAg, the computational studies of water adsorption on (i) the favoured pristine CaAg (010) surface and (ii) already oxygenated and stable CaO@CaAg(010) surfaces were carried out. Using a 2 × 2 surface unit cell (9.38 × 8.12 Å), various initial geometries with different numbers of water molecules were relaxed. The best adsorption energies per water molecule obtained are ~ −0.75 eV for one molecule, and ~ −0.69 eV for 7 and 8 molecules. In all cases, both the surface and water molecule(s) remain intact (Figure 11). The pristine CaAg(010) surface is stable under water vapour.

**Figure 12.**
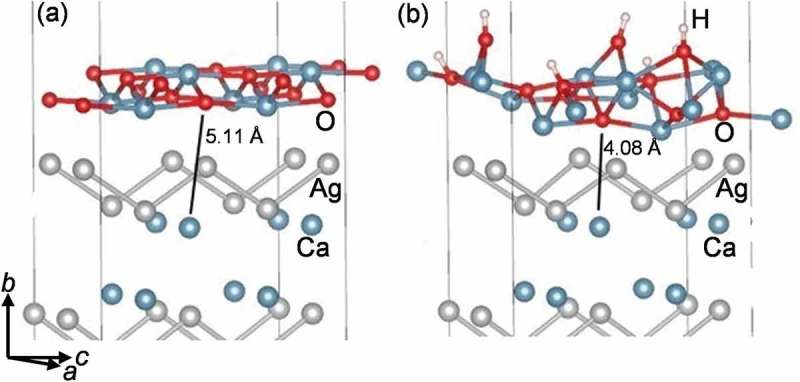
Structural details of the (a) CaO-overlayer and the (b) CaO+H_2_O – overlayer on CaAg (010) surface.

The instability of CaAg under ambient conditions, meaning simultaneous presence of oxygen and water in the atmosphere, can be investigated theoretically by performing adsorption calculations of water molecules on the CaO@CaAg(010) surface. These calculations show that in the presence of water molecules, the passivating layer becomes less ordered and gives rise to locally less strongly bound oxygen atoms. These oxygen atoms will likely be more mobile concerning a diffusion into subsurface areas and thus enable and promote the bulk transformation. Figure 12 illustrates this concept by comparing the shortest distances between surface oxygen atoms and subsurface calcium atoms. In case of CaO@CaAg(010) surface, the shortest distance between an oxygen atom and a subsurface Ca atom is of the order of 5 Å (Figure 12(a)). Upon adsorption on CaO@CaAg(010) layer, water undergoes a spontaneous dissociation into H^+^ and OH^−^ (Figure 12(b)). Moreover, the associated energy of adsorption is −1.2 eV per water molecule, implying sizable surface coverages under exposure to air. Structurally, the dissociated hydrogen associates to a structural oxygen atom whereas the OH^−^ group adsorbs on a Ca-Ca bridge position. These changes are accompanied by the notable downward displacement of a structural oxygen atom, yielding a distance of ~ 4 Å to the nearest subsurface Ca atom (Figure 12(b)). This finding suggests that CaAg is unstable in air, because the adsorption of water molecules on the CaO passivation layer facilitates the downward movement of the layer’s oxygen atoms leading to further decomposition of the bulk.

Concerning the Ca-rich binary compounds, the larger mass gains for under ‘*full conversion*’ conditions compared to ‘*zero conversion*’ mode confirm the observation that they are unstable in air mainly due to the presence of moisture.

## 7. Conclusions

Considering a catalyst for ethylene epoxidation, many factors need to be taken into account, and it is almost impossible to attribute the chemical behaviour and catalytic performance exclusively to one parameter, since the ongoing chemistry is very complex. Nevertheless, combining experimental observations, catalytic performance data, detailed analysis of the compounds concerning the chemical bonding and modelling of molecule adsorption on different surfaces allow to draw reasonable conclusions about the reactivity and catalytic performance of intermetallic Ca–Ag compounds under reaction conditions.

Based on experimental and computational results, the five Ca–Ag intermetallic compounds were divided into three groups: (i) Ag-rich Ca_2_Ag_7_ and CaAg_2_, (ii) equiatomic CaAg, and (iii) Ca-rich Ca_5_Ag_3_ and Ca_3_Ag.

The isotropy of the chemical bonding in Ag-rich binary compounds Ca_2_Ag_7_ and CaAg_2_ and high affinity of Ca towards oxygen favour the oxidation of those compounds under ethylene epoxidation conditions. As a result, Ag particles are formed on complex CaO/Ca(OH)_2_/CaCO_3_ support, providing reasonable ethylene conversion rates (2–3%) and maximum selectivities of 55–65% towards ethylene oxide. In terms of catalysis, these compounds are precursors for an active Ag catalyst with useful catalytic properties due to the complex morphology of the support and its interaction with Ag particles.

With increasing Ca content, the contribution of ionic Ca-Ag interactions to chemical bonding increases. In equiatomic CaAg compound there are preferentially ionic interactions between Ca and Ag atoms and Ag-Ag covalent bonds in (001) plane. Such anisotropy of chemical bonding leads to the preferential cleavage perpendicular to the [010] crystallographic direction. This surface is also able to form stable, ordered and dense CaO-based layer under ethylene epoxidation conditions [35]. This layer hinders the further oxidation of CaAg, but simultaneously prevents the accessibility of Ag, leading to reduced ethylene conversion and low selectivity.

The Ca-rich binary compounds Ca_5_Ag_3_ and Ca_3_Ag are characterized by a significant electron transfer from calcium to silver atoms (Figure 4), leading to the reduced electrophilicity of Ag atoms and, correspondingly, to the decreased selectivity towards desired EO. Furthermore, the conversion of ethylene is very low due to the near-complete coverage of the surface by oxygen-containing layers leading to the absence of accessible Ag atoms on the surface.

The reactivity and oxidation behaviour of the Ca–Ag compounds are strongly dependent on the applied conditions and nature of the used oxidant. Contrary to ethylene epoxidation conditions, the stability of Ca–Ag compounds in air decreases drastically with increasing Ca content. Here, the differences between oxygen atmosphere and exposure to air are ascribed to the water vapour present in air. Additionally, co-feeding of EC promoter has a pronounced effect on the restructuring of the surface and as a result on the corrosion of Ca-rich compounds.

In general, the study on the reactivity of Ca–Ag binary compounds clearly shows that: (i) the different oxidation behaviour is not only determined by thermodynamic properties and the affinity of Ca towards oxygen, (ii) the chemical bonding mainly determines the chemical behaviour of the Ca–Ag compounds and (iii) each component of the system (composition of the atmosphere, temperature, elemental composition of the compound, etc.) plays an important role in the oxidation of Ca–Ag compounds and thereby in their catalytic properties.
