# Supplementary material for: Ca–Ag compounds in ethylene epoxidation reaction
Source: Sci Technol Adv Mater. 2019 Aug 14;20(1):902–16. doi: 10.1080/14686996.2019.1655664 (PMC6758618; doi:10.1080/14686996.2019.1655664)
Supplement: Supplemental Material [file TSTA_A_1655664_SM5719.pdf]

Supporting Information

Ca–Ag compounds in ethylene epoxidation reaction

Iryna Antonyshyn,[a] Olga Sichevych,[a] Alim Ormeci,[a] Ulrich Burkhardt,[a] Karsten Rasim,[a] Sven Titlbach,[b] Marc Armbrüster,[c] Stephan A. Schunk,[b] and Yuri Grin[a]

Table S1 Experimental conditions in the DTA/TG-MS experiments

Mode of experiment	"Zero conversion"[a]	"Full conversion"[b]
Gas ratio, vol. %	O_2:C_2H_4:He = 7:35:58	CO_2:H_2O:C_2H_4:He = 4.7:4.7:32.6:58
Total flow, ml min^{-1}	10	40
Heating/cooling rate, ^{o}C min^{-1}	5	
Maximum *T*, ^{o}C	250	
Isotherm duration, h	10	
Sample mass, mg	30-40	
PSD, µm	20-50	

[a] "mimicking" standard ethylene epoxidation test;

[b] gas mixture was calculated assuming that all 7 vol. % O_2 was converted towards carbon dioxide and water vapour.

Table S2 Crystallographic data on synthesized Ca–Ag binary compounds

Compound	Structure type	Space group	Lattice parameters**			CN (Ag)
			a, Å	*b*, Å	*c*, Å	
Ca_2Ag_7 [55,56]	Yb_2Ag_7	*Cmcm*	6.4770(3)	5.5205(2)	14.089(1)	Ag1: 12 (**8 Ag** + 4 Ca)<br>Ag2: 12 (**8 Ag** + 4 Ca)<br>Ag3: 12 (**8 Ag** + 4 Ca)
$CaAg_2$ [58]	KHg_2	*Imma*	4.691(1)	7.295(2)	8.135(1)	Ag: 10 (**4 Ag** + 6 Ca)
CaAg [58,59]	*α*-TlI	*Cmcm*	4.063(1)	11.460(2)	4.655(1)	Ag: 9 (**2 Ag** + 7 Ca)
Ca_5Ag_3 [60]	Cr_5B_3	*I*4/*mcm*	8.0227(5)	*a*	14.973(2)	Ag1: 10 (all Ca)<br>Ag2: 9 (**1 Ag** + 8 Ca)
Ca_3Ag*	Fe_3C	*Pnma*	7.9820(2)	10.1855(2)	6.8651(2)	Ag: 9 (all Ca)

*The crystal structure was determined in this study for the first time

** Lattice parameters were obtained from present PXRD investigation

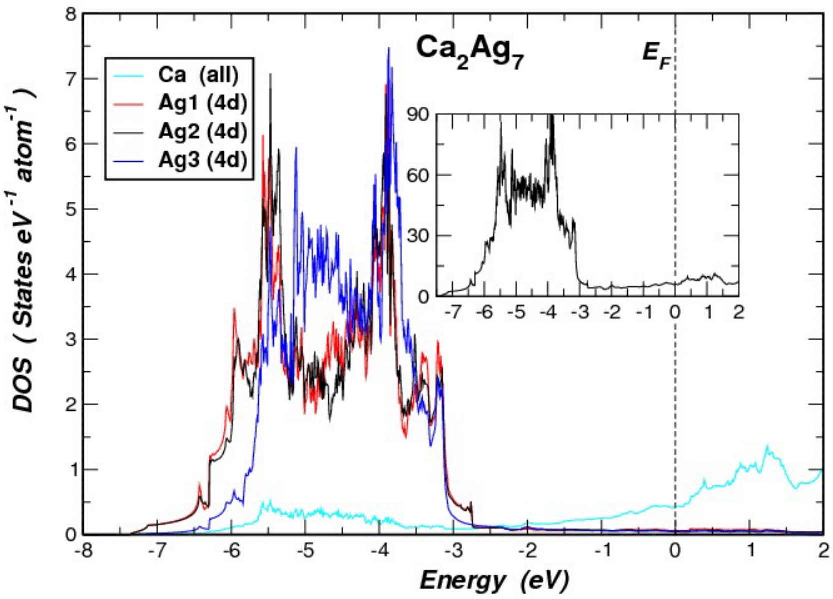
Ca2Ag7
EF
Ca (all)
Ag1 (4d)
Ag2 (4d)
Ag3 (4d)
DOS (States eV-1 atom-1)
Energy (eV)

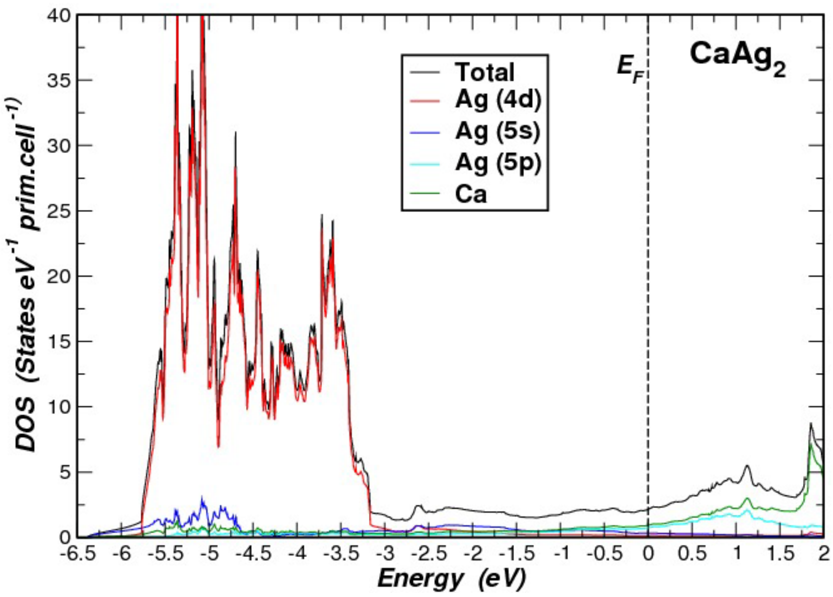
CaAg2
EF
Total
Ag (4d)
Ag (5s)
Ag (5p)
Ca
DOS (States eV-1 prim.cell-1)
Energy (eV)

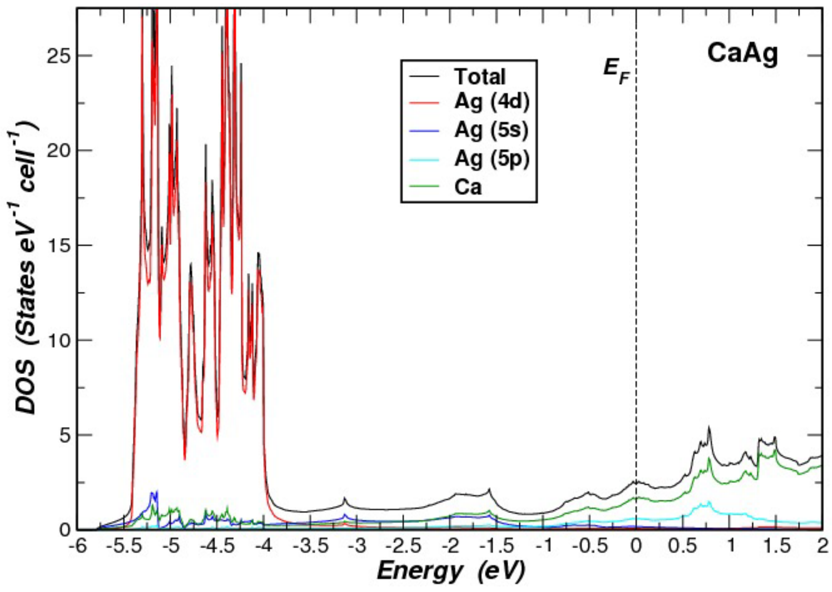
CaAg
EF
Total
Ag (4d)
Ag (5s)
Ag (5p)
Ca
DOS (States eV-1 cell-1)
Energy (eV)

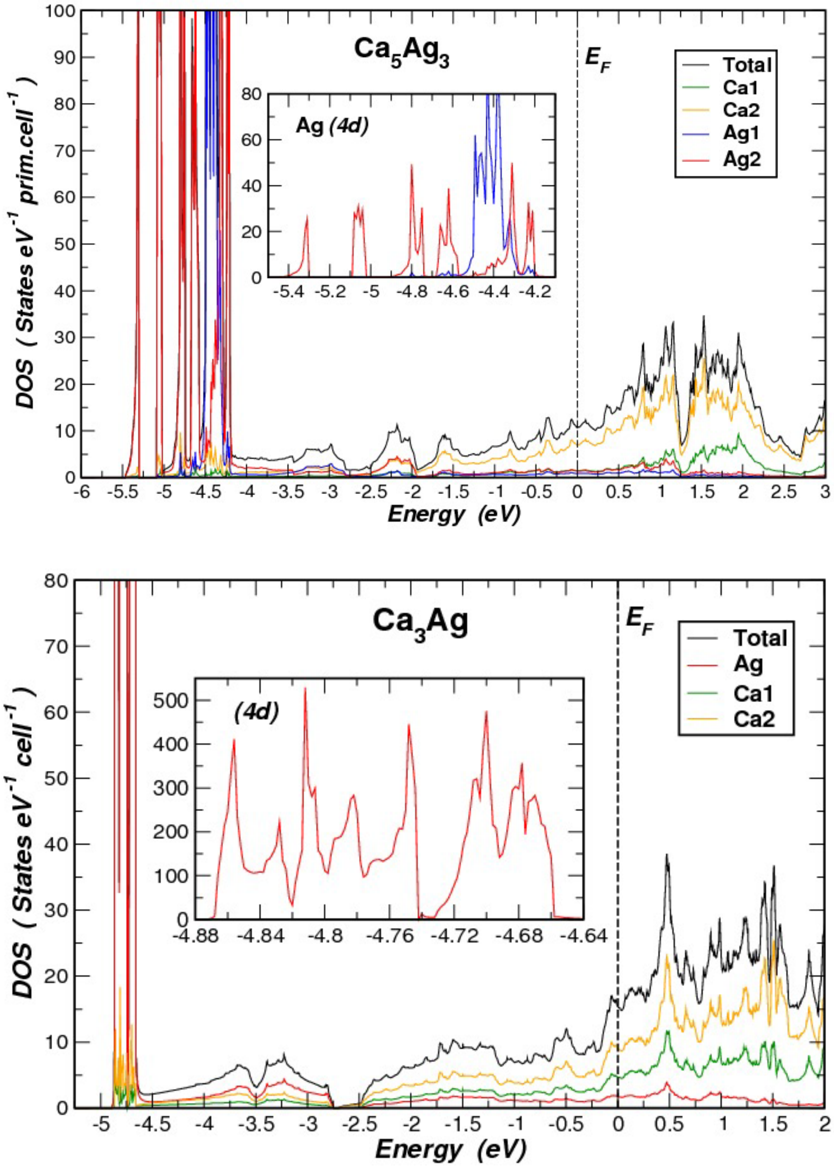

Figure S1. The DOS for Ca–Ag binary compounds calculated by the FPLO method. Total DOSs per eV per primitive cell are shown as insets.

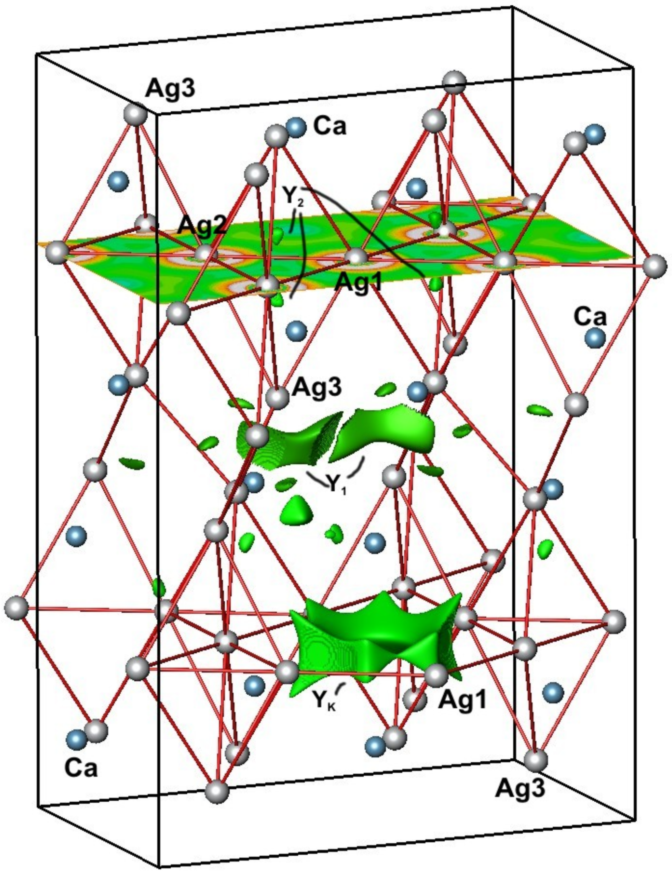

Figure S2. The chemical bonding situation in Ca_2Ag_7. The Ag1 and Ag2 atoms form a slightly distorted Kagome layer (identified by the plane of the contour plot) and the isosurfaces around the ELI-D attractors located in these layers have a six-ring shape (feature labelled as Y_K). The Ag3 and Ca species each form triangular nets, and the main covalent interactions among them are represented by the ELI-D attractors denoted as Y_1. The Y_2 attractors are located inside the Ag_4 tetrahedra formed by Ag1-Ag2-Ag2-Ag3 revealing four Ag-atom interactions (bond population 0.28 electrons). The remaining attractors, not identified in the figure, represent three-Ag-atom bonds with bond populations varying between 0.21 and 0.24 electrons. Ca atom contributions do not exceed 5% level implying strongly polar covalent interactions among the Ca and Ag atoms in this compound.

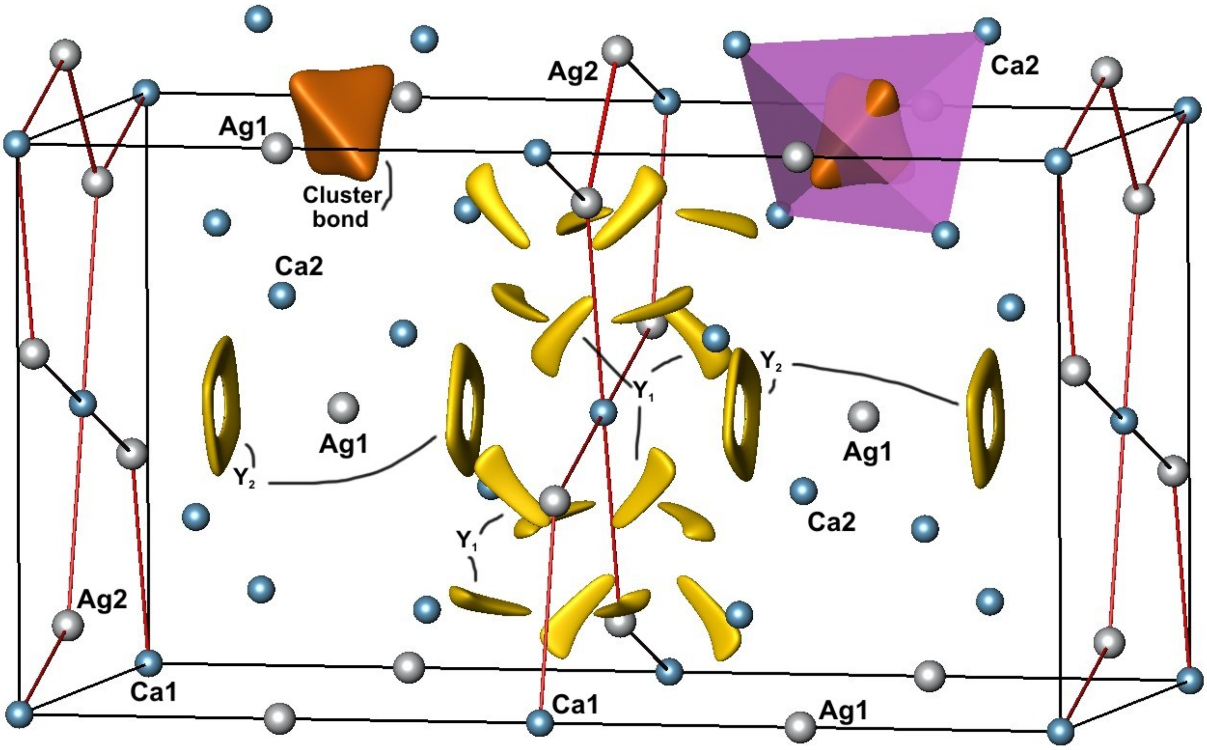

Figure S3. The ELI-D isosurfaces to illustrate the chemical bonding situation in Ca_5Ag_3. The isosurface values are 1.15 (brown) and 1.0 (yellow). The former represents a cluster bond whose population of 6.35 electrons is contributed mainly by 12 atoms (4 Ag1, 4 Ag2 and 4 Ca2). Its attractor is located at the center of the $Ca2_4$ tetrahedron as highlighted in the figure. The bonds labelled by Y_1 (Y_2) are centered on Ag2 (Ag1) with a population of 0.70 (0.24) electrons. Three (two) Ca atoms contribute to the former (latter), each less than 10%.

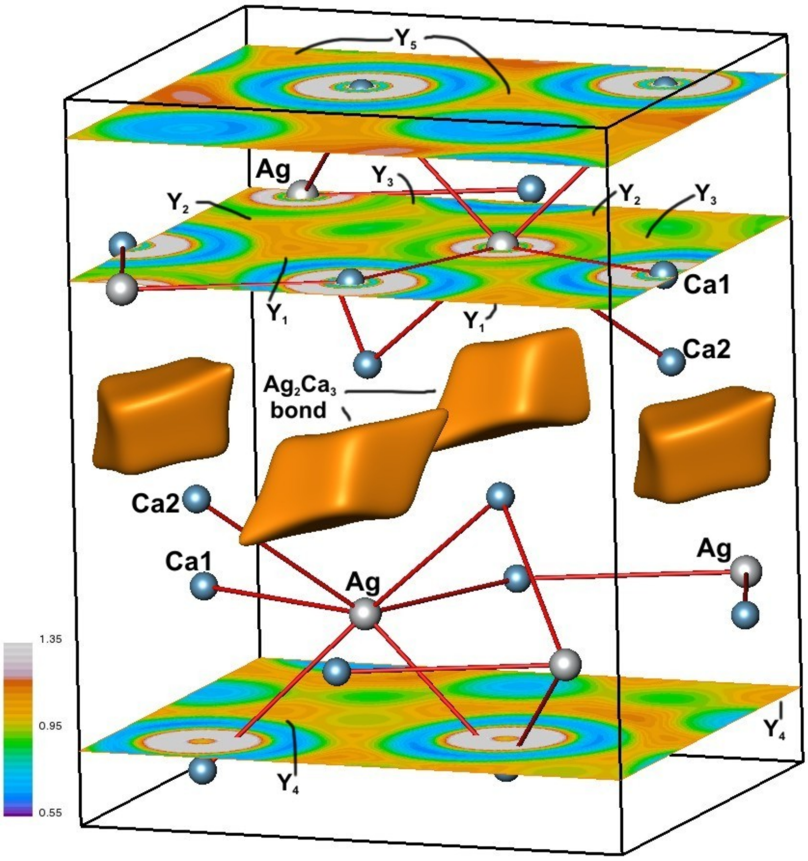

Figure S4. The chemical bonding situation in Ca_3Ag is shown through ELI-D isosurface and two-dimensional contour plots. The isosurfaces (value = 1.14) indicate the five-atom interaction with main contributions coming from two Ag and three Ca atoms. There are two such bonds per each Ag atom and the bond population is 1.85 electrons. The locations of the attractors corresponding to the remaining bonds are denoted by Y_i, i = 1, 5, on the contour plots. Their bond populations vary between 0.21 and 0.60 electrons. In each case the main contributor is a silver atom and three Ca atoms also contribute, each below 10%. Y_1, Y_2 and Y_3 are located in the plane of Ag and Ca1 atoms (y = ¼ and ¾ layers) with a multiplicity of four. The multiplicities of Y_4 and Y_5 are eight, these attractors are distributed over four layers along the *b*-axis.

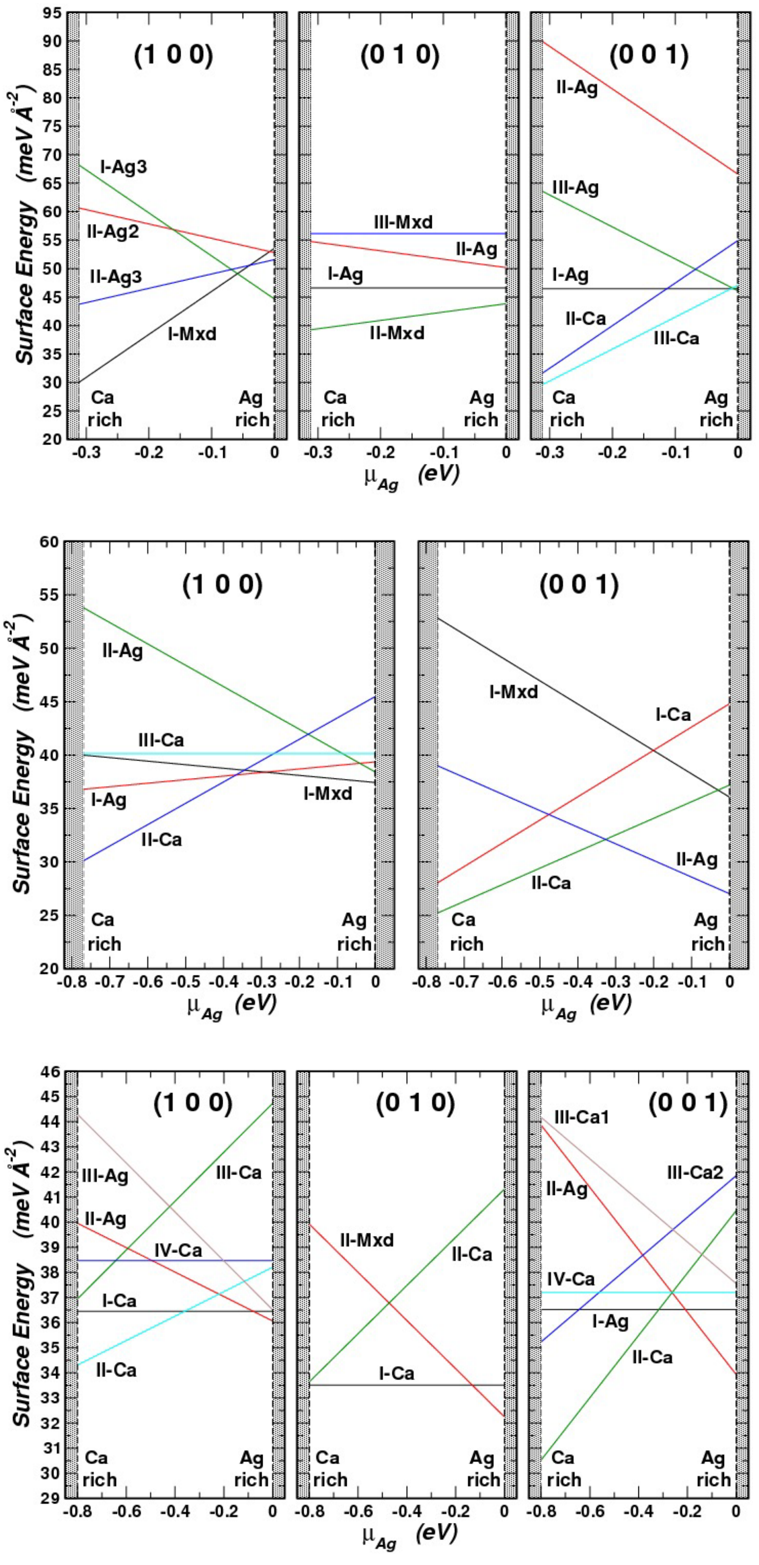

Figure S5. The surface energies of all possible top layer compositions computed for (100), (010) and (001) as a function of Ag chemical potential for Ca_2Ag_7 (top), Ca_5Ag_3 (middle) and Ca_3Ag (bottom). For Ca_5Ag_3 only (100) and (001) surfaces are considered. The Roman numbers stand for the cleavage planes which are shown and defined in Figures S4–S6.

Cleavage planes

Possible cleavage planes perpendicular to [100], [010] and [001] are shown below for Ca_2Ag_7, Ca_5Ag_3, and Ca_3Ag. For each compound and direction the cuts are numbered by Roman numerals. The two resulting surfaces are named according to the cut number and the composition of the top layer. In some cases stoichiometric and symmetric slabs, which are identical, can be constructed from both of these surfaces. The surface energies of such surfaces do not depend on component chemical potentials. The numbers of atoms stated in the figure captions for the corresponding terminating surfaces are per surface unit cell. The labeling of elements (Ag1, Ca2, etc.) follow the notation used in the crystallographic data (Table S2).

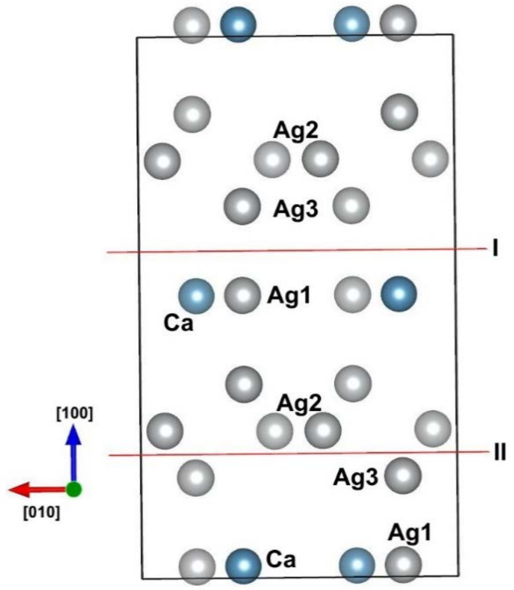
Ag2
Ag3
I
Ag1
Ca
Ag2
[100]
II
[010]
Ag3
Ag1
Ca

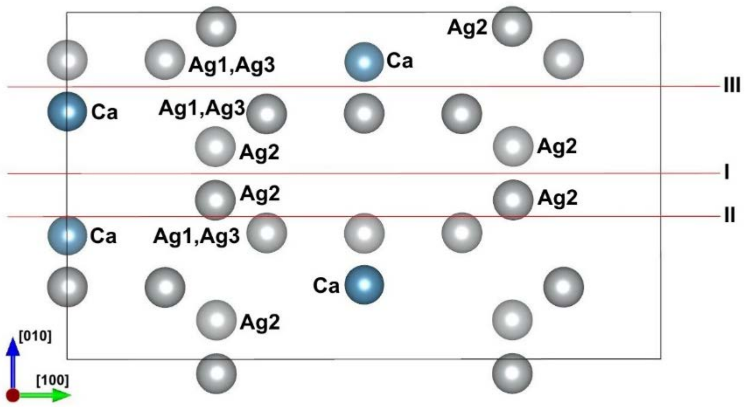
Ag2
Ag1,Ag3
Ca
III
Ca
Ag1,Ag3
Ag2
Ag2
I
Ag2
Ag2
II
Ca
Ag1,Ag3
Ca
Ag2
[010]
[100]

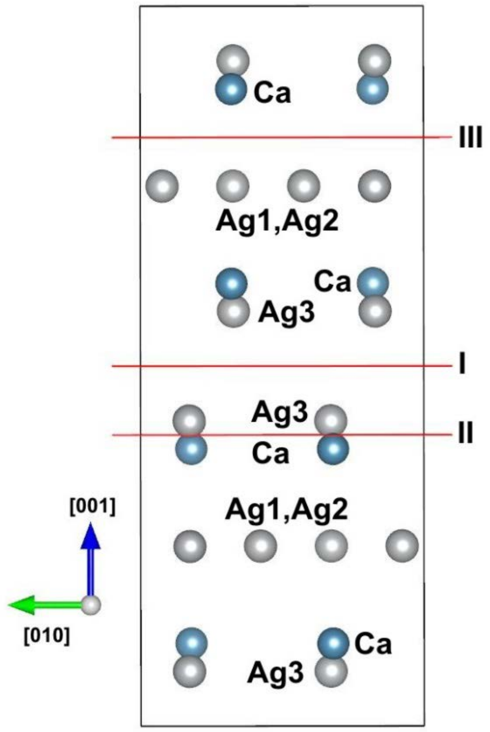
Ca
III
Ag1,Ag2
Ca
Ag3
I
Ag3
II
Ca
[001]
Ag1,Ag2
[010]
Ca
Ag3

Figure S6. Possible cleavage planes perpendicular to [100] (**a**), [010] (**b**) and [001] (**c**) in Ca_2Ag_7. The resulting surfaces are named by using the notation for the surface, the location of the cut (denoted by Roman numerals) and the composition of the surface layer (two possibilities if two surfaces are different). The surfaces are hence referred to as: (100)-I-Mxd (2 Ag1, 4 Ca atoms), (100)-I-Ag3 (4 Ag3 atoms), (100)-II-Ag2 (4 Ag2 atoms) and (100)-II-Ag3 (4 Ag3 atoms) (**a**); (010)-I-Ag (stoichiometric slab), (010)-II-Ag2 (2 Ag2 atoms), (010)-II-Mxd (1 Ag1, 4 Ag3 atoms, 2 Ca atoms) and (010)-III-Mxd (stoichiometric slab) (**b**); and (001)-I-Ag (stoichiometric slab), (001)-II-Ag (4 Ag3 atoms), (001)-II-Ca (2 Ca atoms), (001)-III-Ca (2 Ca atoms) and (001)-III-Ag (2 Ag1, 4 Ag2 atoms) (**c**).

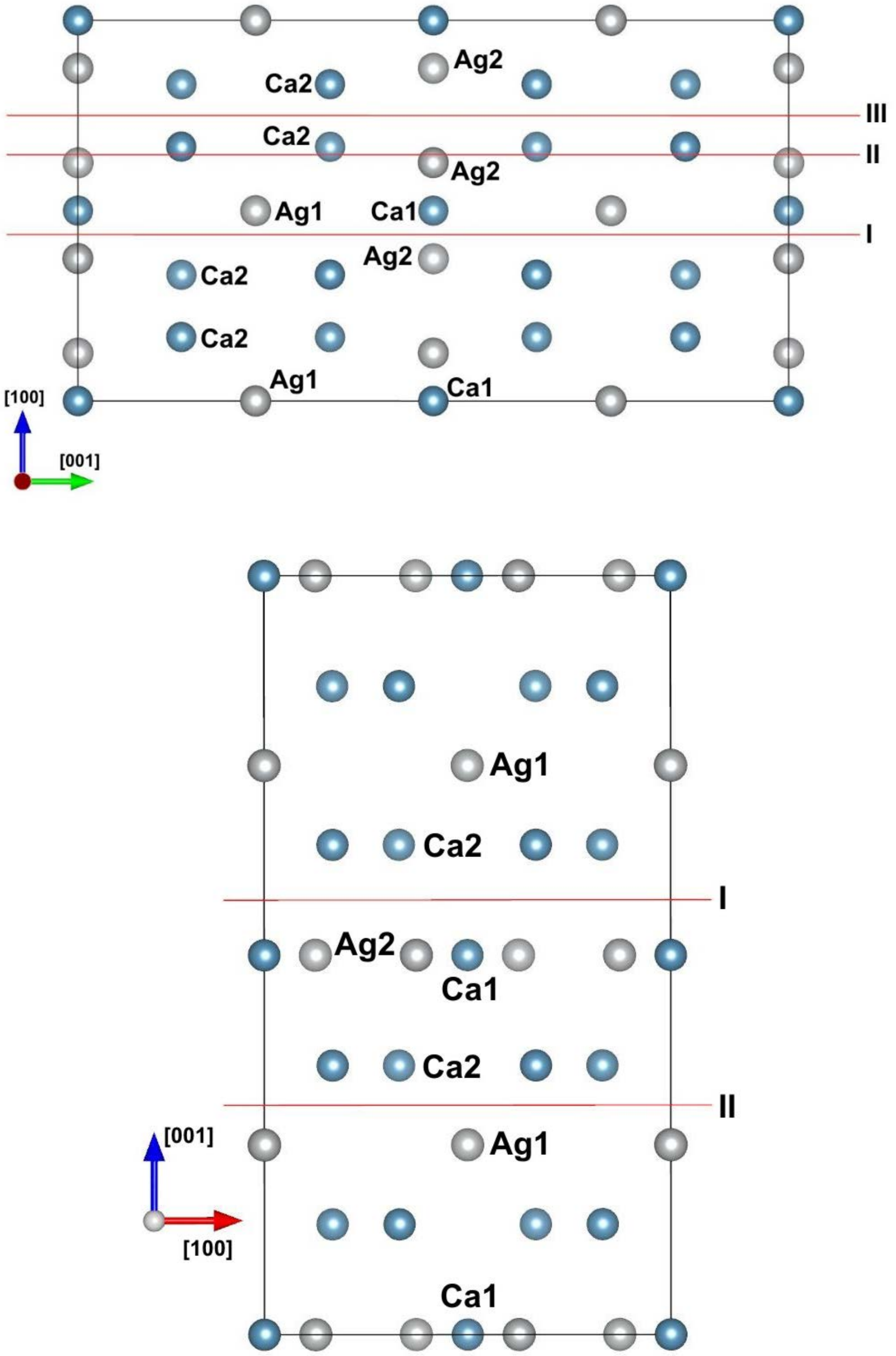

Figure S7. Possible cleavage planes perpendicular to the [100] (**a**) and [001] (**b**) in Ca_5Ag_3. The resulting terminations are denoted as: (100)-I-Ag (2 Ag2 atoms), (100)-I-Mxd (2 Ag1, 2 Ca1 atoms), (100)-II-Ca (4 Ca atoms), (100)-II-Ag (2 Ag2 atoms), (100)-III-Ca (stoichiometric slab) (**a**); (001)-I-Ca (4 Ca2 atoms), (001)-I-Mxd (4 Ag2, 2 Ca1 atoms), (001)-II-Ca (4 Ca2 atoms), (001)-II-Ag (2 Ag1 atoms) (**b**).

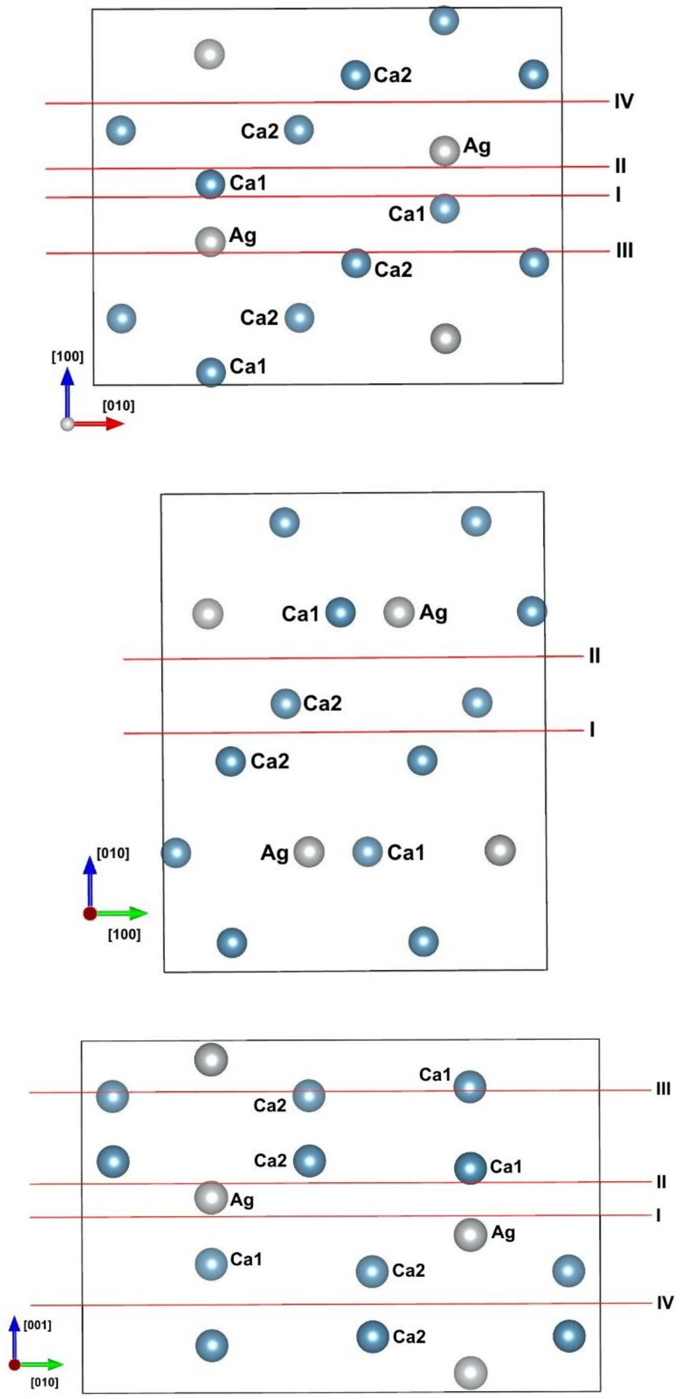

Figure S8. Possible cleavage planes perpendicular to the [100] (**a**), [010] (**b**) and [001] (**c**) in Ca_3Ag. The resulting terminations are denoted as: (100)-I-Ca (stoichiometric slab), (100)-II-Ag (1 Ag atom), (100)-II-Ca (1 Ca1 atom), (100)-III-Ca (2 Ca2 atoms), (100)-III-Ag (1 Ag atom) and (100)-IV-Ca (stoichiometric slab) (**a**); (010)-I-Ca (stoichiometric slab), (010)-II-Mxd (2 Ag, 2 Ca1 atoms) and (010)-II-Ca (2 Ca2 atoms) (**b**); (001)-I-Ag (stoichiometric slab),

(001)-II-Ag (1 Ag atom), (001)-II-Ca (1 Ca1 atom), (001)-III-Ca2 (2 Ca2 atoms), (001)-III-Ca1 (1 Ca1 atom) and (001)-IV-Ca (stoichiometric slab) (**c**).

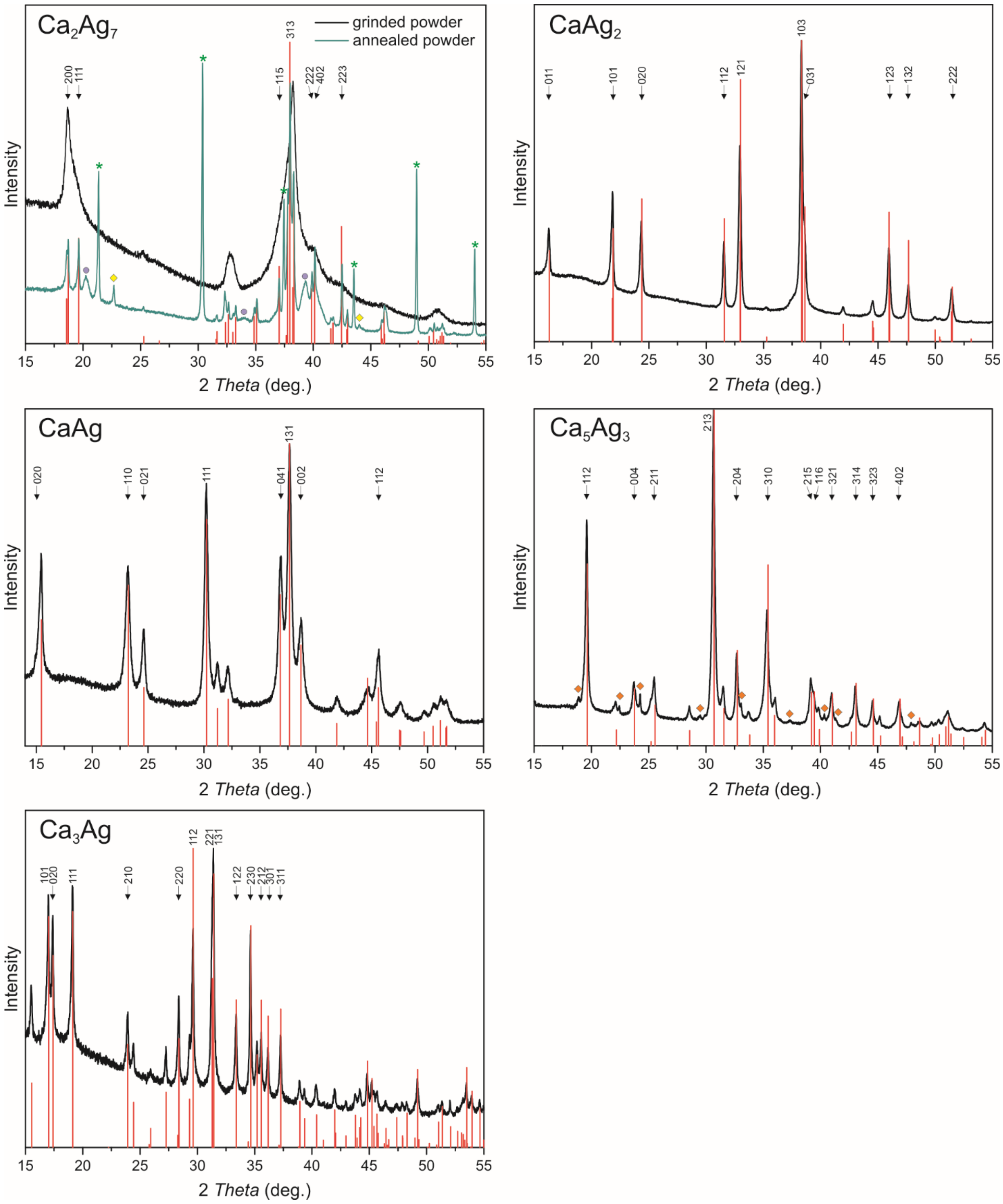

Figure S9. Powder X-ray diffraction patterns of Ca–Ag compounds: experimental patterns (*black line*) together with positions and relative intensity of the peaks for calculated one (*red lines*). The admixture phases are marked by: assumed "Ca_2Ag_9" (*yellow diamonds*), unknown phase (*violet circles*), added standard LaB_6 (*green asterisk*) and binary Ca_3Ag_2 (*orange diamonds*). The *hkl* indexes are marked for the most intense PXRD peaks of the main phases.

The PXRD patterns for Ca_2Ag_7 and $CaAg_2$ are the average of six single measurements, whereas for CaAg, Ca_5Ag_3 and Ca_3Ag only the first patterns are presented due to the high sensitivity of these samples to air.

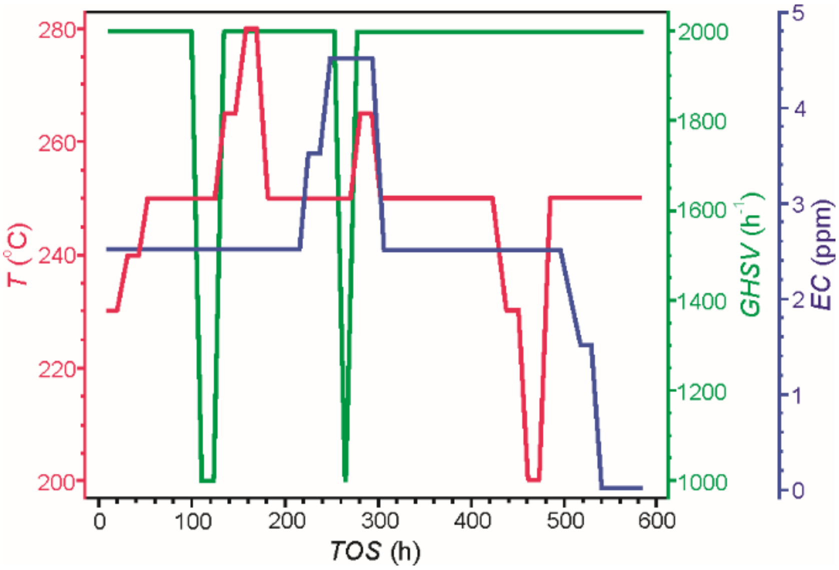

Figure S10. Experimental conditions of standard ethylene epoxidation test.

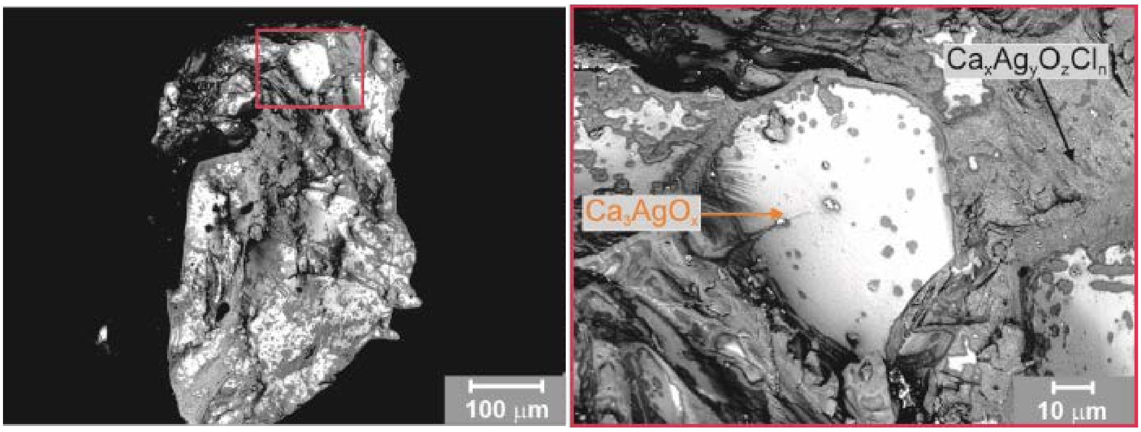

Figure S11. Morphology of Ca_3Ag particles after ethylene epoxidation at elevated temperatures (T_{max} = 350 °C) and presence of ethyl chloride excess (4.5 ppm).
